# Taming unconventional nucleic acid structures: synthesis of acridine- and 1,10-phenanthroline-based G-quadruplex binders

**DOI:** 10.1039/d6ra04126d

**Published:** 2026-07-06

**Authors:** Julián Robin Sárik, István Szatmári, Bálint Lőrinczi

**Affiliations:** a Institute of Pharmaceutical Chemistry, University of Szeged Eötvös u. 6 H-6720 Szeged Hungary pomogacs98@gmail.com szatmari.istvan@szte.hu lorinczi.balint@szte.hu; b HUN-REN SZTE Stereochemistry Research Group, University of Szeged Eötvös u. 6 H-6720 Szeged Hungary

## Abstract

In the past 30 years acridine and 1,10-phenanthroline ring systems have become more widespread in the investigation of novel G-quadruplex binding compounds. Starting from the well-known BRACO19 and PhenDC3 these biologically active scaffolds have undergone several major structural changes to improve the affinity and selectivity of these noncanonical DNA and RNA structures. The library of these molecules is dominated by symmetrical derivatives, however recently asymmetrical analogues have also found their way to be the focus of several research groups. Furthermore, the modification of the 1,10-phenanthroline ring system has also been investigated and has led to derivatives with competent G-quadruplex binding capabilities. The aim of this review is to provide a comprehensive look into the chemistry behind these molecules and to investigate the most widely used methods to achieve these compounds.

## Abstract 2

In the past 30 years acridine and 1,10-phenanthroline ring systems have become more widespread in the investigation of novel G-quadruplex binding compounds.

## Graphical abstract

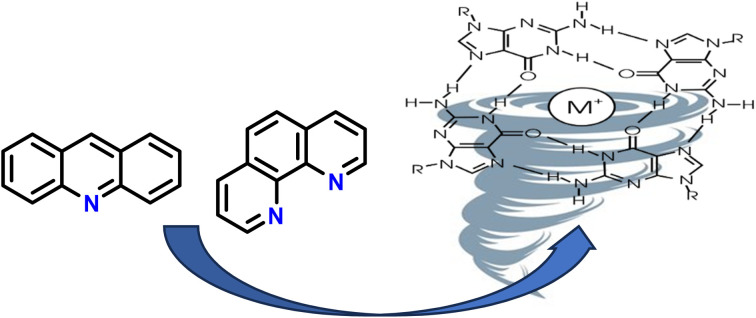

## 1 Introduction

G-quadruplexes (Gqs) are noncanonical secondary structures formed in the guanine rich sequences of DNA and RNA. Interacting *via* Hoogsteen hydrogen bonds, four guanine bases form a flat and cyclic G-quartet (also known as G-tetrad), two or more of which subsequently create the three-dimensional Gq structure with π–π stacking interactions.^1,2^ In their central channel the presence of metal cations (typically Na^+^ and K^+^) also serves as a stabilizing factor in the formation of these systems.^3,4^ Gqs have been observed in several regions of the human genome with major biological functions: telomeres, oncogenic promoters (such as Bcl-2, c-MYC, c-KIT, k-RAS, *etc.*) and RNA 5′-untranslated regions (5′-UTR).^5–9^ Due to their role in cell development Gqs are especially abundant in tumor cells.^10,11^ Considering this, the development of small molecules targeting these nucleic acid structures proved to be an important part of expanding the library of anticancer agents.^12^ It is interesting to mention that the property of DNA and RNA to form Gqs cannot be regarded as a human-specific phenomenon. The presence of these structures has also been investigated in viral nucleic acid, *e.g.*, in HIV, West Nile and Influenza A virus as well,^13–15^ thus antiviral utilization can be a potential consideration for these compounds as well.

**Fig. 1:**
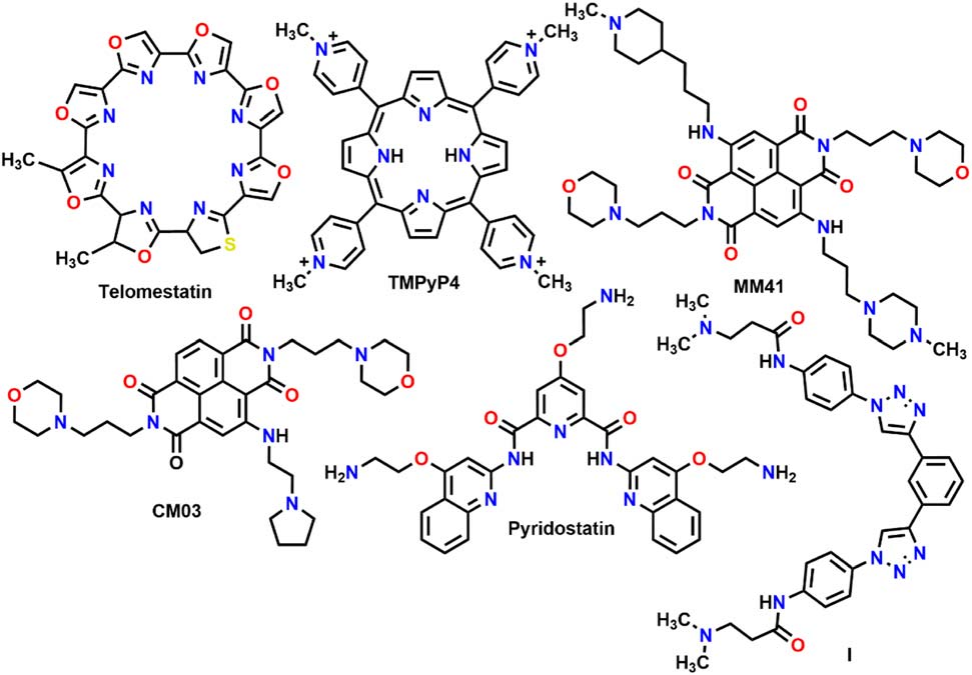
The structure of the aforementioned Gq binding agents.

Several studies have provided some key principles that can be beneficial in the design of new Gq binding molecules. Planar, aromatic or heteroaromatic ring systems capable of forming strong π–π stacking and electrostatic interactions have been proven to be effective at stabilizing the G-tetrads. Moreover, the presence of cationic centers or positive charges in the molecule can further enhance this property.^16–19^ Numerous compounds fulfilling these requirements have been synthesized in the last few decades. Notable ones are the oxazole ring-based macrocycles (telomestatin and its derivatives),^20,21^ porphyrin derivatives (TMPyP4),^22,23^ naphthalene diimide derivatives (MM41, CM03)^24,25^ and different symmetrical non-fused aromatic systems (pyridostatin, compound I) (Fig. 1).^26,27^

**Fig. 2:**
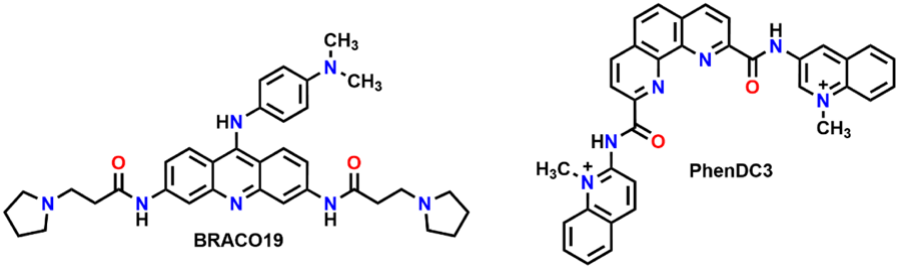
The structure of BRACO19 and PhenDC3.

Another small, albeit all the more important part of the Gq binding molecular library is the acridine and phenanthroline based compounds. According to the above-mentioned principles these ring systems possess strong π–π stacking and electrostatic interactions increasing their selectivity towards Gqs. Furthermore, acridine and 1,10-phenanthroline – the latter being considered a strong Lewis-base – have potent chelating properties thus are able to form strong coordination complexes. Considering the presence of Na^+^ and K^+^ cations in Gq structures the stabilizing effect of the acridine and 1,10-phenanthroline ring systems is further improved.^28,29^ Some of their representative compounds are one of the first investigated Gq binding agents (BRACO19 and PhenDC3) and are used in the investigation of Gq structures to this day (Fig. 2).^30,31^

While PhenDC3 has superior selectivity to Gqs over duplex DNA,^32^ BRACO19 was found out to be subpar in this regard (compared to recent compounds),^30^ however this unfavourable property can be remedied through structural modifications. The aim of this review is to provide a comprehensive history of the synthesis of the different acridine and phenanthroline derivatives thus shedding light on the importance of these compounds.

## 2 Acridine derivatives

### 2.1 ymmetric acridine derivatives

**Scheme 1:**
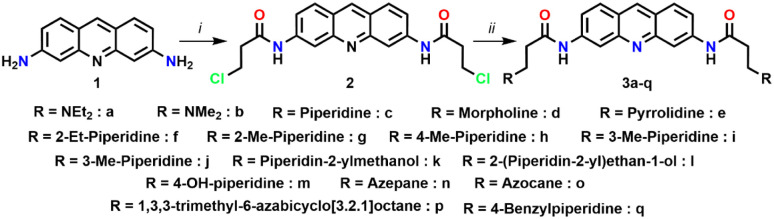
Synthesis of BRACO19 precursors. (i) 3-Chloropropionyl chloride, 80 °C, 3–4 h; (ii) R_2_NH, NaI, EtOH, reflux, 2–4 h.

One of the first synthesized and certainly a well-known Gq binding acridine derivative is BRACO19. The precursor molecules of this compound (without the substituent at C-9) were synthesized and evaluated by Harrison *et al.* in 1999. Using 3-chloropropionyl chloride and different secondary amines the research group synthesized 16 different molecules (Scheme 1).^33,34^

**Scheme 2:**
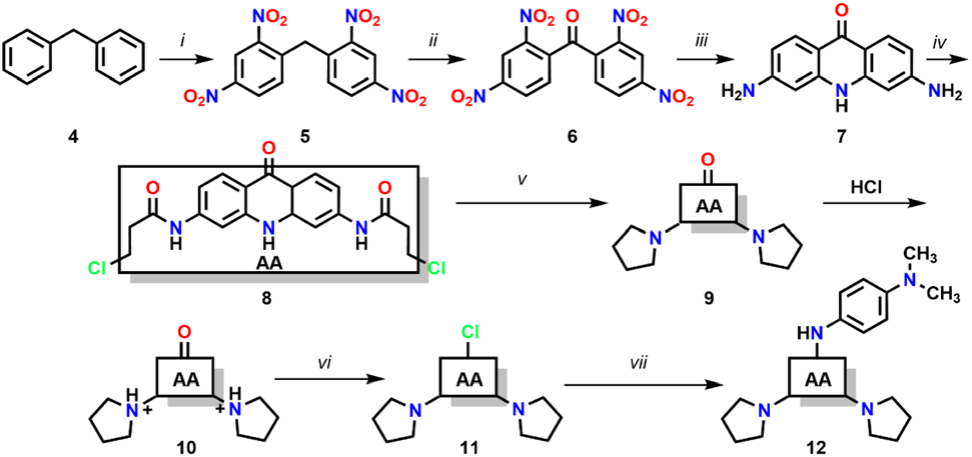
Synthesis of BRACO19. (i) KNO_3_/H_2_SO_4_; (ii) CrO_3_, AcOH, reflux; (iii) Zn/HCl, 90–100 °C; (iv) chloropropanoyl chloride, reflux; (v) pyrrolidine, NaI, EtOH, reflux; (vi) POCl_3_, reflux; (vii) *N*,*N*-dimethylbenzene-1,4-diamine, CHCl_3_, reflux.

Based on the acquired telomerase inhibition IC_50_ values and further computational calculations, the fine-tuning of the molecules was continued with the pyrrolidine derivative by Read *et al.* Starting from 1,1′-methylenedibenzene BRACO19 was acquired at the end of an eight-step synthetic procedure (Scheme 2).

**Fig. 3:**
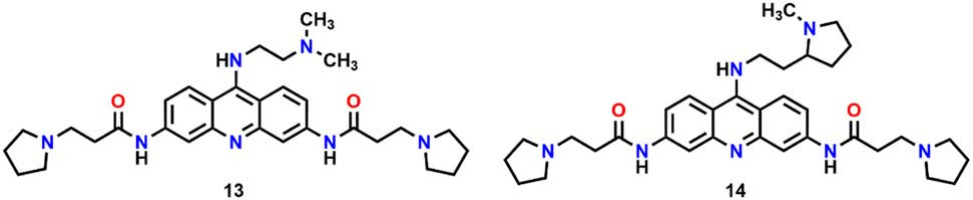
The most efficient Gq binding acridine derivatives.

The introduction of the *N*,*N*-dimetilaminophenylamine group to the C-9 position resulted a ten-fold increase in their Gq affinities while also keeping a strong selectivity towards the quadruplex structure.^30^ The structure–activity relationship of BRACO19 and its derivatives was further investigated by Harrison *et al.* Different C-9 substitutions and 3,6,9-; 2,6,9- and 2,7,9-regioisomers were evaluated based on Gq and duplex DNA binding affinities. The synthesis of these compounds was carried out based on the procedure previously described by Read *et al.* Out of the regioisomers the 3,6,9-analogues were found out to be generally the most effective and 2,7,9-isomers the least potent. Based on the experimental values it can also be surmised that the presence of the anilino group at C-9 was not a necessity as aliphatic side chains also showed comparable affinities. Compounds introducing a cationic moiety in the C-9 position provided the most compelling quadruplex binding values (Fig. 3).^35^ Similar phenomenon was observed in case of the amide side chain.^36^ These findings were further reinforced in a later study by Harrison *et al.* by investigating the C-9 acridone derivative and the previously described amide regioisomers.^37^

**Scheme 3:**
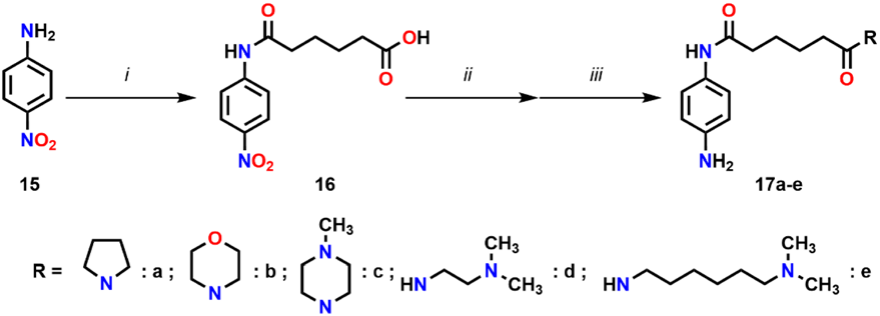
Synthesis of the 9-anilino side-chains. (i) Adipic anhydride, 1,4-dioxane, reflux; (ii) RNH_2_/R_2_NH, ethyl chloroformate, TEA, DCM, 0 °C; (iii) 10% Pd/C, ammonium formate, MeOH, r.t.

The structure–effect relationship of BRACO19 derivatives were extended to analogues with different functions in the C-9 position and in the C-3 and C-6 amide side chain in the following years. Using amidoanilines as a replacement of *N*,*N*-dimetilaminophenylamine by Schultes *et al.*, the synthesis of numerous BRACO19 analogues were carried out. The 9-anilino side-chains were prepared using the following method (Scheme 3), the end product was achieved *via* coupling reaction of the aniline amine moiety as described above.^30,38^ Compelling telomerase inhibition and Gq binding affinity were observed in case of compounds coupled with 17d and 17e while 17a–c showed mixed results.

**Scheme 4:**
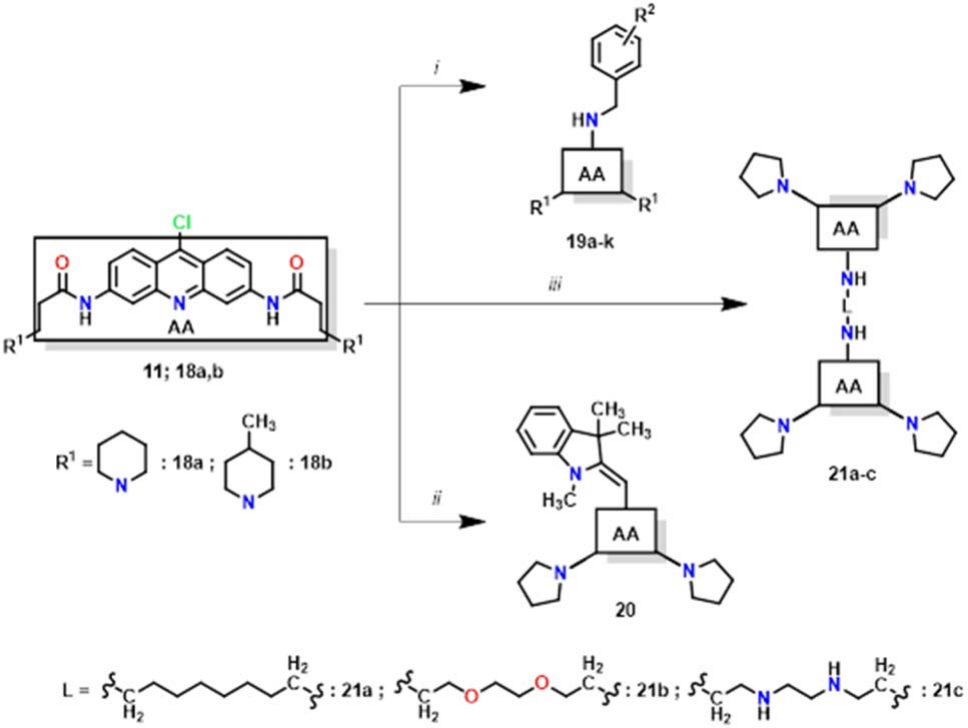
Synthesis of acridine derivatives 19a–k, 20 and 21a–c. (i) Amine, DIPEA, ACN, reflux, 28 h; (ii) (from 11): 2-methylene-1,3,3-trimethylindoline, CHCl_3_, 60 °C, 6 days; (iii) (from 11): H_2_NLNH_2_, phenol, 100 °C, 2 h.

With the 9-anilino side-chain thoroughly investigated the aim was shifted to the synthesis of benzylamine and other unique C-9 substituted analogues. The first of these derivatives was the benzylamine containing analogue investigated by Martins *et al.* The research group described the synthesis of numerous compounds containing different *N*-heterocyclic functions at the end of the C-3 and C-6 side-chains however until the final step the methods utilized align with previously reported literature routes (Scheme 4). The C-9 substituted derivatives 19a–k were achieved using the 9-chloro intermediate *via* nucleophilic substitutions using the corresponding functionalized benzylamines (Table 1).^39^ Following this research project a unique pH sensing chimera was investigated by Percivalle *et al.* Starting from 9-chloro intermediate of BRACO-19 and reacting it with 2-methylene-1,3,3-trimethylindoline fluorescent cyanine dye 20 was obtained with great affinity to oncogene promoters (Scheme 4).^40^ Also starting from the often-mentioned intermediate 11 Fu *et al.* achieved the synthesis of acridine dimers (Scheme 4). Reacting 11 with different aliphatic diamines using phenol as solvent the desired products 21a–c were acquired with moderate yields.^41^

**Table 1:** Functional groups of acridine compounds 19a–k

Comp.	19a	19b	19c	19d	19e	19f	19g	19h	19i	19j	19k
R^2^	4-N(Me)_2_	3,4-F,F	3,5-diOMe	3-Me	3-F-5-F_3_C	3,5-diF_3_C	3,4-F,F	3,5-diOMe	3-Me	3-Me	4-N(Me)_2_
N	0	1	1	1	1	1	1	1	1	1	0
R^1^	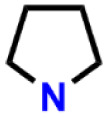	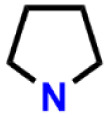	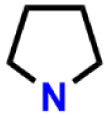	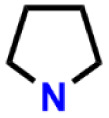	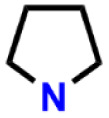	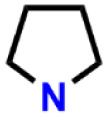	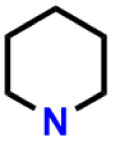	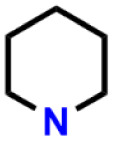	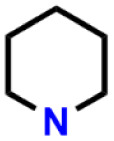	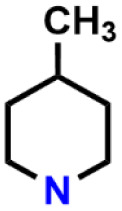	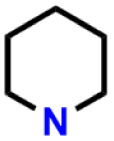

**Scheme 5:**
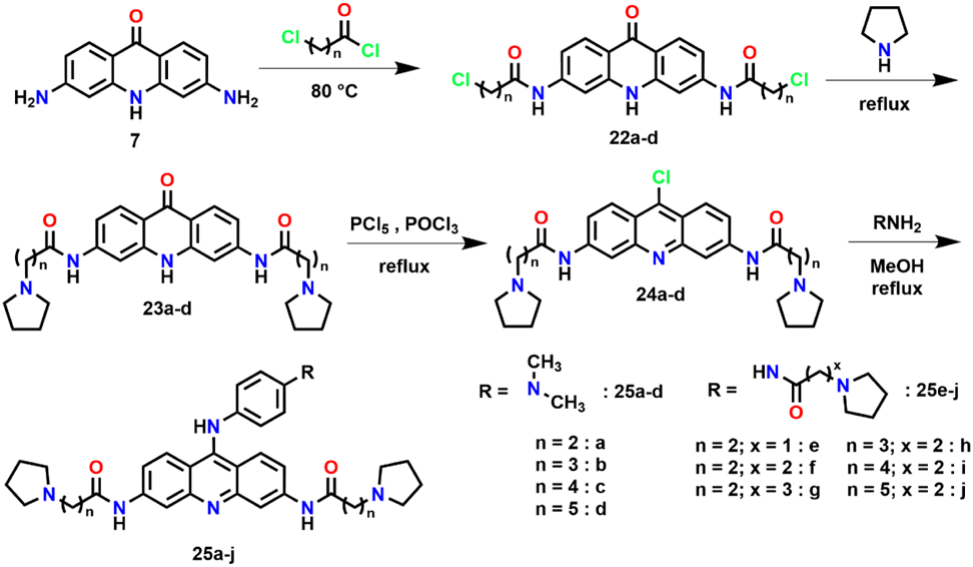
Synthesis of acridine derivatives containing modified side-chains.

Remaining still in the realm of BRACO-19 derivatives the effect of the length of the amide side-chain in the C-3 and C-6 positions was investigated for further structure–effect relationship establishment. To achieve this 7 was reacted with the corresponding carboxylic acid halides containing chlorine at the end of the aliphatic chains to achieve the amide derivatives (Scheme 5). The end products 25a–j were obtained using the original method with minor optimizations applied.^42^ Apart from the dimethylaminoaniline other aniline derivatives containing the same amide function as the C-3 and C-6 side-chain were used as substituents at C-9. A different route was also described by Moore *et al.* which involves the synthesis of an azide analogue from 7 from which the C-9 functionalization was first accomplished followed by the formation of the C-3 and C-6 side-chain after azide reduction. Generally, more favorable yields were provided by the latter method thus it was used to achieve the 3-(pyrrolidin-1-yl)propanamide derivatives which showed lower reactivity utilizing the original method.

**Scheme 6:**
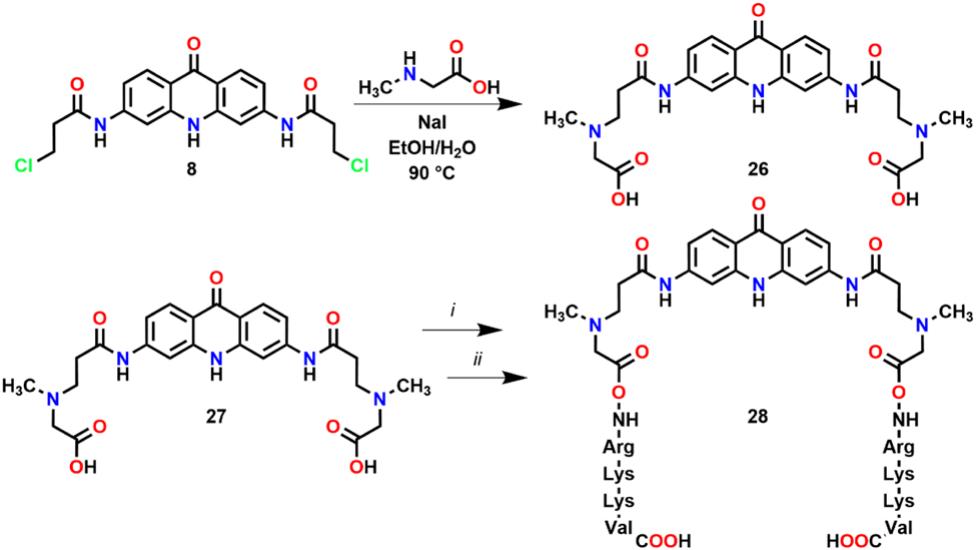
Synthesis of acridine oligopeptide derivatives. (i) Peptide, PyBOP (benzotriazol-1-yloxytripyrrolidinophosphonium hexafluorophosphate), 1-HOBt, DIPEA, DMSO, 50 °C; (ii) TFA/TIS/H_2_O.

Starting from a precursor of BRACO-19 but achieving vastly different results Ladame *et al.* reported the synthesis of a unique acridine derivative containing oligopeptides at the amide side-chains (Scheme 6). To obtain this compound intermediate 11 was reacted with *N*-methylglycine to introduce carboxylic acid function to the side-chains.^43^ After using a coupling reaction to form the peptide bond with two oligopeptides sharing the same protecting group and then deprotecting the oligopeptides utilizing acidic hydrolysis 28 was obtained with excellent yields. This method involves the usage of resin-bound peptides and according to the researchers it appears to be general. However, it should be mentioned that acridine (and 1,10-phenanthroline) peptide derivatives didn't play major role in the extension of Gq binding molecular library despite the presence of this method.

**Scheme 7:**
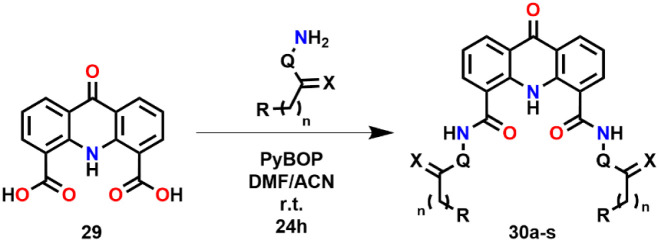
Synthesis of 4,5 substituted acridone derivatives.

Other amide derivatives not functionalized at C-9 position were investigated by Cuenca *et al.* As mentioned previously the 3,6 substitution is not a necessity to achieve Gq binding properties, thus the research group chose the 4,5 dicarboxyl acridone analogue to investigate the effect of diverse side chain lengths and substituents on biological activity (Scheme 7). Aniline derivatives containing various aminoalkyl amide functions were chosen as candidates and after a coupling reaction involving 29 as starting material and PyBOP as reagent compound 30a–s was achieved.^44^ Using 4-carboxyl acridone asymmetric amide derivative including *N*-(4-aminophenyl)-3-(pyrrolidin-1-yl)propenamide as the side-chain was achieved with the same method as well (Table 2).

**Table 2:** Compounds 30a–s and their functional groups

Q	X	*n*	R	Compound
*p*-Ph	=O	2	Pyrrolidin-1-yl	30a
*p*-Ph	=O	2	Morpholino	30b
*p*-Ph	=O	2	Dimethylamino	30c
*p*-Ph	=O	2	4-Methylpiperazin-1-yl	30d
*p*-Ph	=O		Piperidin-1-yl	30e
*p*-Ph	=O	2	Cyclohexylamino	30f
*p*-Ph	=O	2	1*H*-imidazole-1-yl	30g
*p*-Ph	=O	2	4-Oxo-4-(pyrrolidin-1-yl)	30h
*p*-Ph	H_2_	3	Pyrrolidin-1-yl	30i
*p*-Ph	H_2_	3	Morpholino	30j
*p*-Ph	H_2_	3	Dimethylamino	30k
*p*-Ph	H_2_	3	4-Methylpiperazin-1-yl	30l
*p*-Ph	=O	1	Pyrrolidin-1-yl	30m
*p*-Ph	=O	3	Pyrrolidin-1-yl	30n
*p*-Ph	=O	4	Pyrrolidin-1-yl	30o
*m*-Ph	=O	2	Pyrrolidin-1-yl	30p
1-Methyl-1*H*-pyrrol-3-yl^a^	n.a.	3	Pyrrolidin-1-yl	30q
3-(dimethylamino)propyl	n.a.	n.a.	n.a.	30r
*p*-Ph^b^	=O	2	Pyrrolidin-1-yl	30s

a Alkyl function with R is located in position C-5.

b Compound with carboxyl-function only at position C-4 (asymmetric derivative).

**Scheme 8:**
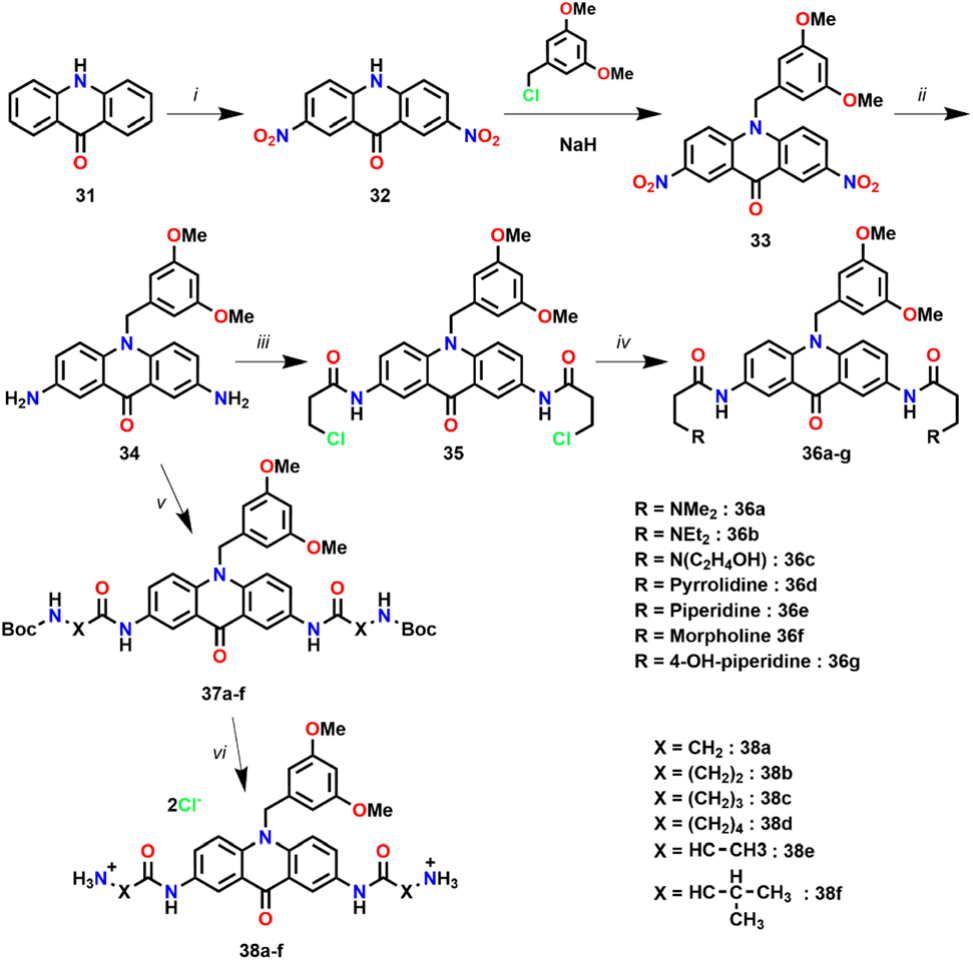
Synthesis of *N*-substituted acridone derivatives. (i) HNO_3_/AcOH, HNO_3_/H_2_SO_4_; (ii) Na_2_S·9H_2_O, EtOH; (iii) 3-chloropropanoyl chloride, 4-methylmorpholine, THF, reflux; (iv) R_2_NH, KI, EtOH, reflux; (v) Boc-AA (AA = amino acid), DIC, 1-HOBt, THF, r.t.; (vi) HCl/1,4-dioxane.

Another 9-acridone regio isomer was investigated by Gao *et al.* while also abandoning the C-9 functionalization in favor of introducing a dimethoxybenzyl group to the nitrogen of the acridine ring system (Scheme 8). To achieve this, 9-acridone was nitrated, then reacted with 3,5-dimethoxybenzylchloride in the presence of sodium hydride to obtain 33. After reduction of the nitro moiety utilizing disodium sulfide 34 underwent a nucleophilic acyl substitution reaction using 3-chloropropanoyl chloride resulting in 35. After another nucleophilic substitution involving alkyl and alkoxyl secondary amines, 36a–g were achieved as the end products of the first method.^45^ As part of the second method, starting from 34 Gao *et al.* also introduced amino acids to the amide side chain utilizing coupling reactions with protected α–δ amino acids, followed by acidic hydrolysis of the Boc protecting group to acquire 38a–f.

**Scheme 9:**
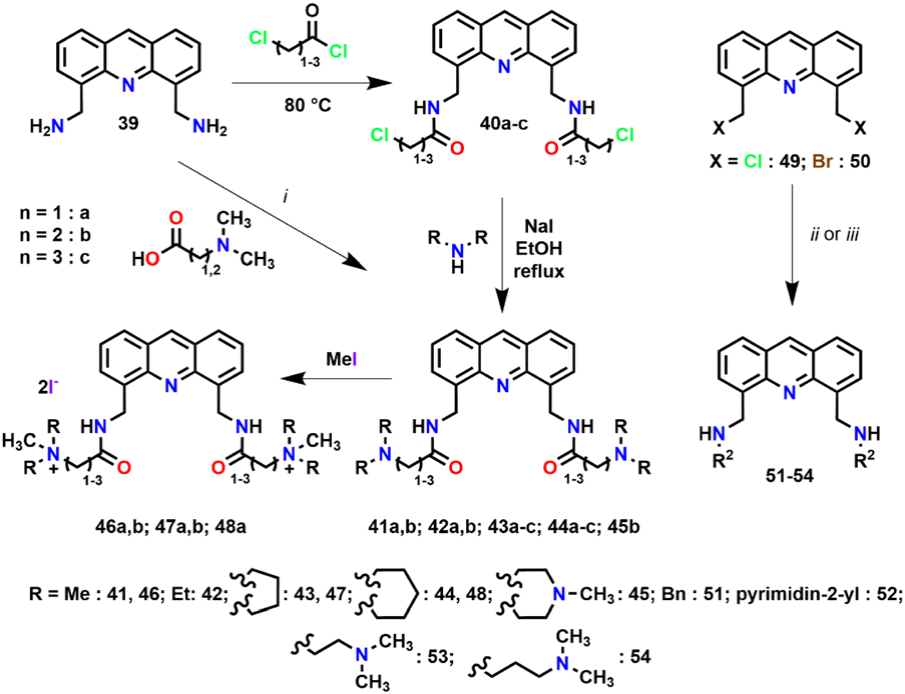
Synthesis of 4,5-acridone amide derivatives. (i) 1-HOBT, DCC, Et_3_N, DCM, r.t., 72 h; (ii): 49, benzylamine (in case of 51) or 2-(dimethylamino)ethylamine (in case of 52), DMSO, r.t., 3 h or 6 h; (iii) 50, 2-aminopyrimidine (in case of 53), K_2_CO_3_, TBAHS, DCM, 50 °C, 24 h.

With the 9-acridone derivatives thoroughly investigated, the original acridine ring system was put into highlight again. Similar compounds as previously described by Reed and Harrison *et al.* were synthesized starting from acridine-4,5-diyldimethanamine (Scheme 9). To achieve the desired compound Laronze-Cochard *et al.* described two routes. The first one involves the usage of chloro-acyl halides to acquire the amide derivatives 40a–c followed by a subsequent nucleophilic substitution with the corresponding secondary amines obtaining 42–45a–c. As the second route a simple coupling reaction utilizing α- and β-amino acids lead to 41a–b. Unfortunately, comparisons between the efficiency of the two routes cannot be made as no overlapping compounds were synthesized using the different methods.^46^ Secondary amine analogues were also achieved by Laronze-Cochard *et al.* starting from the dichloro- (to achieve 51 and 52) and dibromo- (to achieve 53) derivatives 49 or 50 and using aryl and aminoalkyl primary amines.

**Scheme 10:**
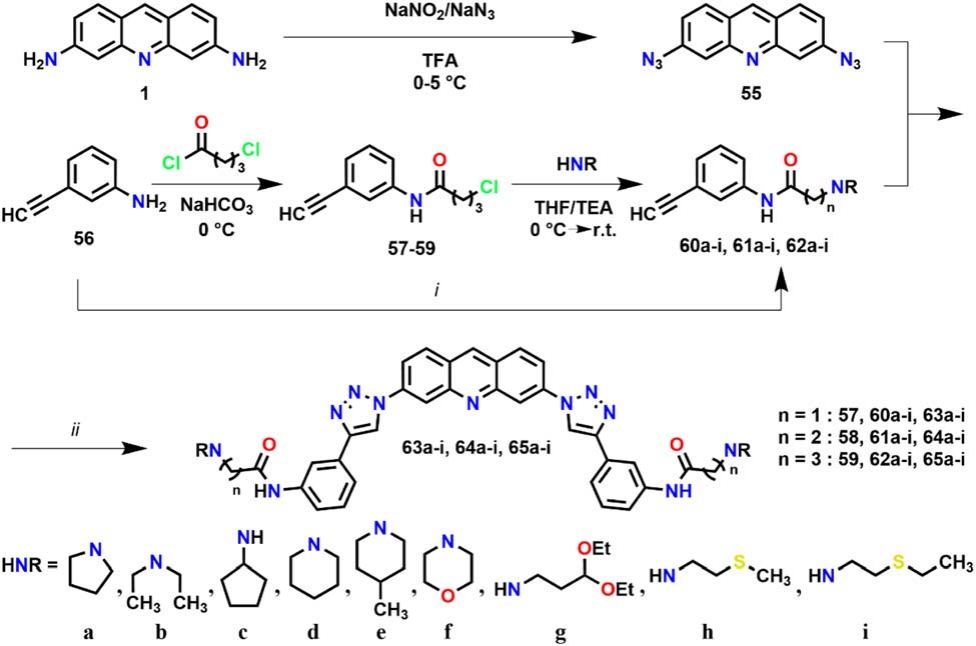
Synthesis of triazolo-acridine derivatives. (i) Chloroacetyl chloride (in case of a) or chloropropanoyl chloride (in case of b), RNH_2_, THF/TEA, 0 °C to r.t.; (ii) (+)-Na-ascorbate, CuSO_4_·5H_2_O, tBuOH, r.t.

To achieve effective Gq binding acridine analogues the popular “click” reaction can be used as demonstrated by Sparapani *et al.* The research group investigated the same 2,6-diaminoacridin originally used for the synthesis of BRACO19 precursors as starting material (Scheme 10). To obtain the necessary azide compound a diazotation reaction was utilized on 1 followed by a substitution with sodium azide. The alkyne component of the triazole ring formation was prepared from 3-ethynylaniline by introducing an aminoalkyl amide function to the amine moiety. The end products 63–65a–i were achieved following the reaction of 55 and 60–62a–i utilizing sodium-ascorbate and copper(ii)-sulphate as catalysts, with yields spreading from high 40 s to low 80 s.^47^

**Scheme 11:**
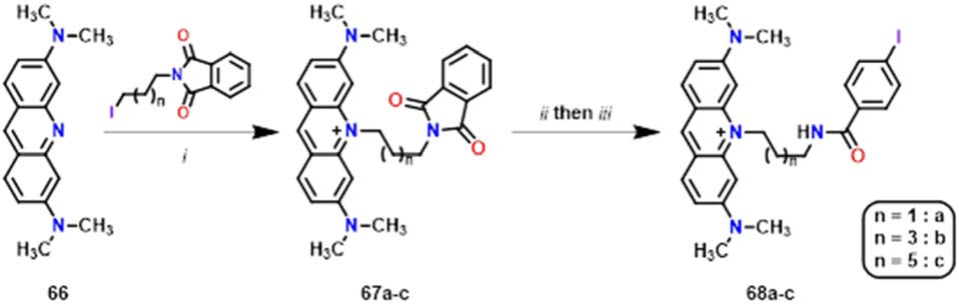
Synthesis of acridinium derivatives. (i) *p*-xylene, N_2_, reflux, 24 h; (ii-1) hydrazine hydrate, dry MeOH, N_2_, reflux, 5 days. (2) HCl (37%), NaOH (4M); (iii-1) DMF, DIPEA, 0 °C, 30 min. (2) Tetrafluorophenyl-4-iodobenzoate, r.t., overnight.

While Gao *et al.* investigated the preparation of *N*-substituted derivatives on 9-acridone Pereira *et al.* and Carvalho *et al.* have taken a different approach. Starting from 2,7-dimethylamino acridine (66) *N*-substituted acridinium compounds were achieved using *N*-iodoalkyl phthalimides (Scheme 11). Following this reaction the phthalimide group was substituted to a primary amine moiety utilizing hydrazine hydrate. The desired products 68a–c were obtained through an amidation reaction using tetrafluorophenyl-4-iodobenzoate with acceptable yields.^48,49^

### 2.2 Asymmetric acridine derivatives

**Scheme 12:**
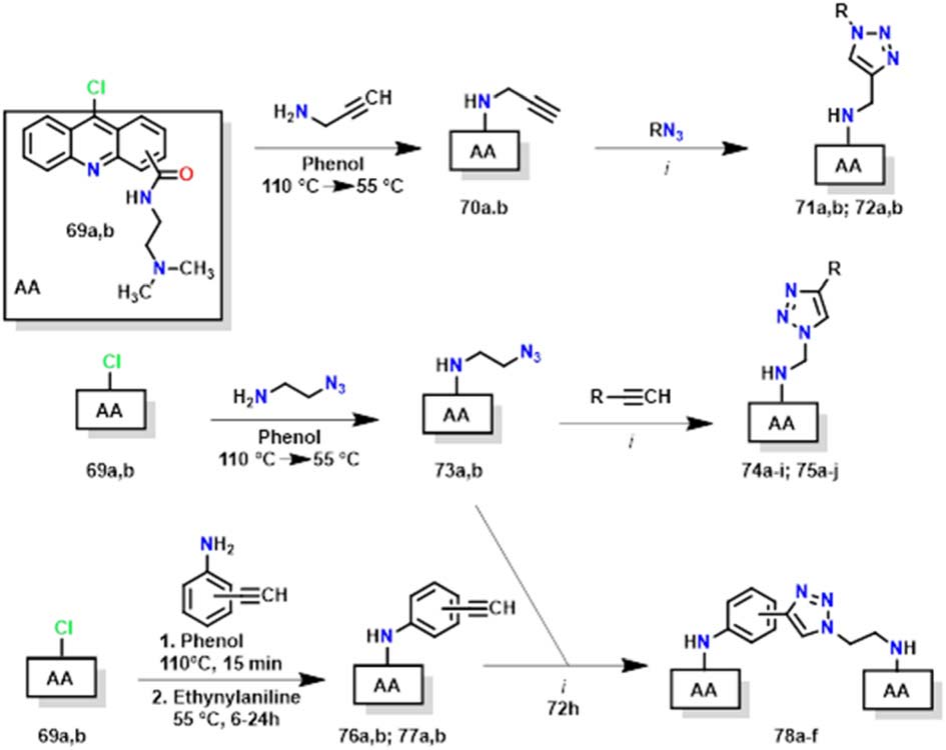
Acridine derivatives synthesized *via* “click” reaction. (i) CuSO_4_, Na-ascorbate, *t*-BuOH/H_2_O, r.t., 24 h.

The “click” reaction was also utilized by Howell *et al.* in the synthesis of C-9 substituted acridine amides. Two routes, both starting from the 9-chloro acridine amide were investigated by the research group to achieve the desired triazole compounds (Scheme 12). As the first method propargylamine was reacted with 69a,b using phenol as the solvent followed by the “click” reaction involving benzyl-azide derivatives. Only a small library of molecules (71a,b, 72a,b) could be prepared this way as the intermediate 70a,b proved to be rather unstable making reactions with it difficult to carry out.

The second route proved to be more fruitful as compound 73a,b – acquired from a similar reaction as 70a,b, only utilizing 2-azidoethan-1-amine instead of propargylamine – was easier to work with. Consequently, a higher number of triazole derivatives (74/75) were achieved with the reaction of 73a,b and aryl- or hydroxyl alkynes. Despite the difficulties of the first route nearly identical yields were achieved comparing the two methods.^50^

Acridine dimers linked through the triazole ring (78a–f) were also synthesized by Howell *et al*. To achieve these compounds 69a,b were reacted with ethynyl-aniline derivatives to yield 76a,b and 77a,b. The desired acridine dimers were achieved following the subsequent reaction of the more stable intermediate 73a,b and 76a,b/77a,b. The acquired yields were fairly spread out ranging from mid 50 s to 100 (Table 3).

**Table 3:** Functional groups of “click” reaction-derived acridine derivatives

Compound	Amide^a^	R
69a	C-3	—
69b	C-4	—
70a	C-3	—
70b	C-4	—
71a	C-3	Benzyl
71b	C-4	Benzyl
72a	C-3	4-Bromobenzyl
72b	C-4	4-Bromobenzyl
73a	C-3	—
73b	C-4	—
74a	C-3	Phenyl
75a	C-4	Phenyl
74b	C-3	2-Bromophenyl
75b	C-4	2-Bromophenyl
74c	C-3	3-Chlorophenyl
75c	C-4	3-Chlorophenyl
74d	C-3	4-Bromophenyl-
75d	C-4	4-Bromophenyl-
74e	C-3	4-Chlorophenyl
75e	C-4	4-Chlorophenyl
74f	C-3	4-Methylphenyl
75f	C-4	4-Methylphenyl
74g	C-3	4-Trifluoromethylphenyl
75g	C-4	4-Trifluoromethylphenyl
74h	C-3	Hydroxymethyl
75h	C-4	Hydroxymethyl
75i	C-4	Carboxylate
74i	C-3	2-Aminobenzamido
75j	C-4	2-Aminobenzamido
		*Ortho*- or *meta*-ethynylaniline
76a	C-3	*Ortho*
76b	C-4	*Ortho*
77a	C-3	*Meta*
77b	C-4	*Meta*
78a	C-4 + C-4	*Meta*
78b	C-3 + C-4	*Meta*
78c	C-4 + C-4	*Ortho*
78d	C-3 + C-4	*Ortho*
78e	C-4 + C-3	*Meta*
78f	C-3 + C-3	*Meta*

a Position of the amide sidechain on the acridine structure.

**Scheme 13:**
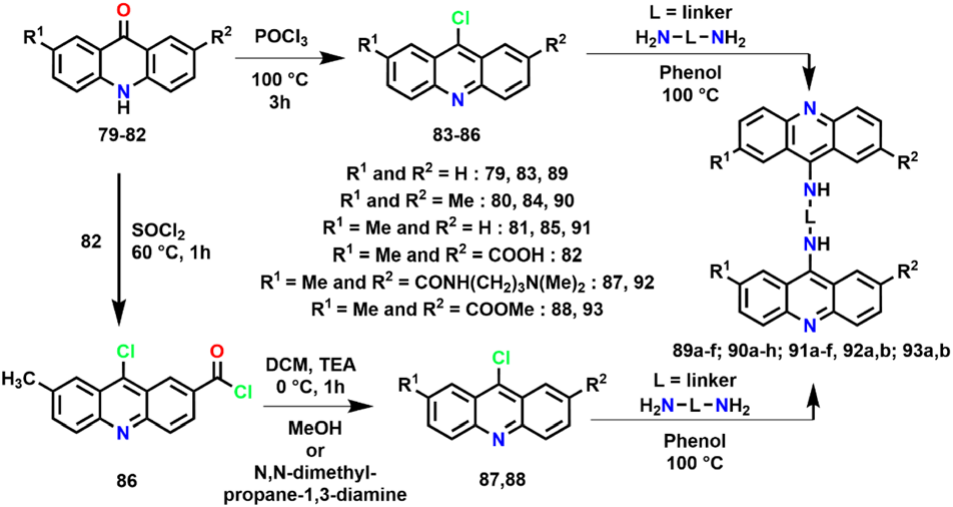
Synthesis of asymmetric and symmetric acridine dimers.

Acridine dimers were also synthesized by Kuang *et al.* however, different linkers were utilized then the ones described by Howell *et al.* The starting material 79–82 was acquired from an Ullmann–Goldberg condensation reaction between anthranilic acid analogues and substituted bromobenzenes and their subsequent ring closure using concentrated sulfuric acid. Following a chlorine substitution with phosphoryl chloride the end products 89a–f, 90a–h and 91a–f were achieved utilizing diaminoalkyl secondary amines and diaminoalkyl ethers (NH_2_-l-NH_2_, Scheme 13).^51^ The carboxylic acid derivative 82 was further functionalized utilizing thionyl chloride to achieve a more reactive acyl chloride followed by an amidation or esterification reaction to achieve 87 and 88. The dimers 92a,b and 93a,b were subsequently formed from 87 and 88 using the same conditions as previously mentioned. The methods used by Kuang *et al.* have already been described in literature (Table 4).^30,41^

**Table 4:** Acridine derivatives and their functional groups and linking groups

R^1^	R^2^	L	Compound
–H	–H	–NHCH_2_CH_2_NHCH_2_CH_2_NH–	89a
–CH_3_	–CH_3_	–NHCH_2_CH_2_CH_2_N(CH_3_)CH_2_CH_2_CH_2_NH–	90a
–CH_3_	–H	–NHCH_2_CH_2_CH_2_N(CH_3_)CH_2_CH_2_CH_2_NH–	91a
–H	–H	–NHCH_2_CH_2_CH_2_N(CH_3_)CH_2_CH_2_CH_2_NH–	89b
–CH_3_	–CH_3_	–NHCH_2_CH_2_CH_2_NHCH_2_CH_2_NHCH_2_CH_2_CH_2_NH–	90b
–H	–H	–NHCH_2_CH_2_CH_2_NHCH_2_CH_2_NHCH_2_CH_2_CH_2_NH–	89c
–CH_3_	–CH_3_	–NHCH_2_CH_2_OCH_2_CH_2_OCH_2_CH_2_NH–	90c
–CH_3_	–H	–NHCH_2_CH_2_OCH_2_CH_2_OCH_2_CH_2_NH–	91b
–H	–H	–NHCH_2_CH_2_OCH_2_CH_2_OCH_2_CH_2_NH–	89d
–CH_3_	–CH_3_	–NHCH_2_CH_2_CH_2_NH–	90d
–H	–H	–NHCH_2_CH_2_CH_2_NH–	89e
–CH_3_	–CH_3_	–NHCH_2_CH_2_CH_2_CH_2_NH–	90e
–CH_3_	–H	–NHCH_2_CH_2_CH_2_CH_2_NH–	91c
–CH_3_	–CH_3_	–NHCH_2_CH_2_CH_2_CH_2_CH_2_NH–	90f
–CH_3_	–H	–NHCH_2_CH_2_CH_2_CH_2_CH_2_NH–	91d
–CH_3_	–CH_3_	–NHCH_2_CH_2_CH_2_CH_2_CH_2_CH_2_NH–	90g
–CH_3_	–H	–NHCH_2_CH_2_CH_2_CH_2_CH_2_CH_2_NH–	91e
–CH_3_	–CH_3_	–NHCH_2_CH_2_CH_2_CH_2_CH_2_CH_2_CH_2_NH–	90h
–CH_3_	–H	–NHCH_2_CH_2_CH_2_CH_2_CH_2_CH_2_CH_2_NH–	91f
–H	–H	–NHCH_2_CH_2_CH_2_CH_2_CH_2_CH_2_CH_2_NH–	89f
–CH_3_	Amide	–NHCH_2_CH_2_OCH_2_CH_2_OCH_2_CH_2_NH–	92a
–CH_3_	Amide	–NHCH_2_CH_2_CH_2_NHCH_2_CH_2_NHCH_2_CH_2_CH_2_NH–	92b
–CH_3_	Ester	–NHCH_2_CH_2_OCH_2_CH_2_OCH_2_CH_2_NH–	93a
–CH_3_	Ester	–NHCH_2_CH_2_CH_2_NHCH_2_CH_2_NHCH_2_CH_2_CH_2_NH–	93b

**Scheme 14:**
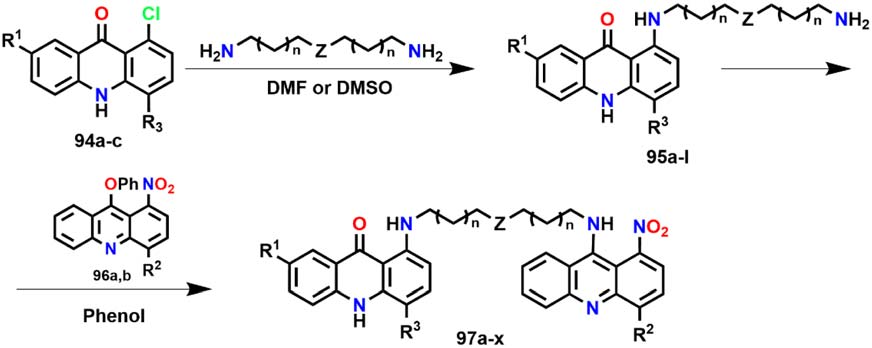
Synthesis of polyamine linked nitroacridine derivatives.

Remaining still at bis-acridine derivatives Paluszkiewicz *et al.* described the synthesis of nitroacridine-acridone dimers. To achieve these compounds polyamine linkers were utilized. Bearing structural similarity to the above-mentioned dimers 97a–x were synthesized using the previously described literature methods difference being the first step where DMF or DMSO as solvent or neat conditions were applied (Scheme 14).

**Scheme 15:**
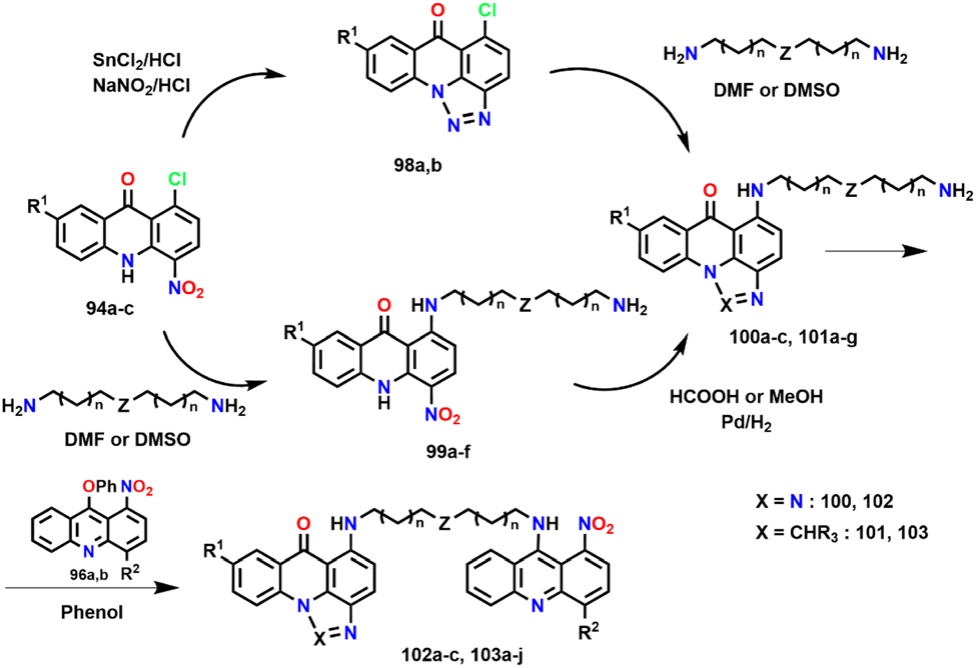
Synthesis of azole type polyamine linked nitroacridine derivatives.

Paluszkiewicz *et al.* also reported novel acridine ring systems, namely imidazoacridinone and triazoloacridinone (Scheme 15). The 1-nitroacridone derivative 94a–c was utilized in a two-step process. To achieve the imidazoacridone ring the polyamine linker was first connected to 94a–c followed by the formation of the five membered ring using formic acid or methanol and a subsequent reduction. As for the triazole analogue, the ring formation occurred as the first step utilizing a diazotation and reduction reaction achieving 100a–c after attaching the polyamine linker. The synthesis of 102a–c and 103a–k concluded with the substitution reaction involving 96a,b and 100a–c/101a–g yielding the nitroacridine-acridone dimers 102a–c and 103a–j as the end products. This step also differs from literature as a wide range of temperatures were utilized depending on the ring system and the substituents (Table 5).^52^

**Table 5:** Acridone-1-nitroacridine compounds (97a–x) of Scheme 15 and imidazo- (103a–j) and azoloacrideone-1-nitroacridone compounds (102a–c) of Scheme 16

Compounds	R^1^	R^2^	R^3^	*n*	Z
97a	OH	H	NO_2_	1	–N(CH_3_)–
97b	OH	H	NO_2_	1	-(Piperazinyl(1,4))-
97c	H	H	CH_3_	1	–N(CH_3_)–
97d	H	H	CH_3_	0	–NH–
97e	H	H	CH_3_	1	-(Piperazinyl(1,4))-
97f	H	CH_3_	CH_3_	0	(CH_2_)_2_NH(CH_2_)_2_
97g	H	CH_3_	CH_3_	1	-(Piperazinyl(1,4))-
97h	OH	CH_3_	NO_2_	1	–N(CH_3_)–
97i	OH	CH_3_	NO_2_	1	-(Piperazinyl(1,4))-
97j	OH	CH_3_	NO_2_	1	–NH–
97k	H	CH_3_	CH_3_	1	–NH–
97l	H	CH_3_	CH_3_	1	–N(CH_3_)–
97m	H	CH_3_	CH_3_	0	–NH(CH_2_)_2_NH–
97n	H	H	NO_2_	1	–N(CH_3_)–
97o	H	CH_3_	NO_2_	1	–N(CH_3_)–
97p	H	CH_3_	NO_2_	1	-(Piperazinyl(1,4))-
97q	H	H	NO_2_	1	-(Piperazinyl(1,4))-
97r	H	CH_3_	NO_2_	0	–NH(CH_2_)_2_NH–
97s	H	H	NO_2_	0	–NH(CH_2_)_2_NH–
97t	H	H	CH_3_	1	–N(CH_3_)–
97z	H	CH_3_	NO_2_	1	–NH(CH_2_)_2_NH–
97x	H	H	NO_2_	1	–NH(CH_2_)_2_NH–
102a	OH	H		1	-(Piperazinyl(1,4))-
102b	H	H		1	-(Piperazinyl(1,4))-
102c	OH	H		1	–N(CH_3_)–
103a	OH	CH_3_	H	1	-(Piperazinyl(1,4))-
103b	OH	H	H	1	-(Piperazinyl(1,4))-
103c	H	H	H	1	–N(CH_3_)–
103d	H	H	H	0	–NH(CH_2_)_2_NH–
103e	H	H	H	1	-(piperazinyl(1,4))-
103f	OH	H	H	1	–N(CH_3_)–
103g	OH	CH_3_	H	1	–N(CH_3_)–
103h	OCH_3_	H	H	1	-(Piperazinyl(1,4))-
103i	H	CH_3_	H	1	-(Piperazinyl(1,4))-
103j	OH	H	CH_3_	1	-(Piperazinyl(1,4))-
103k	H	CH_3_	H	1	–N(CH_3_)–

**Scheme 16:**
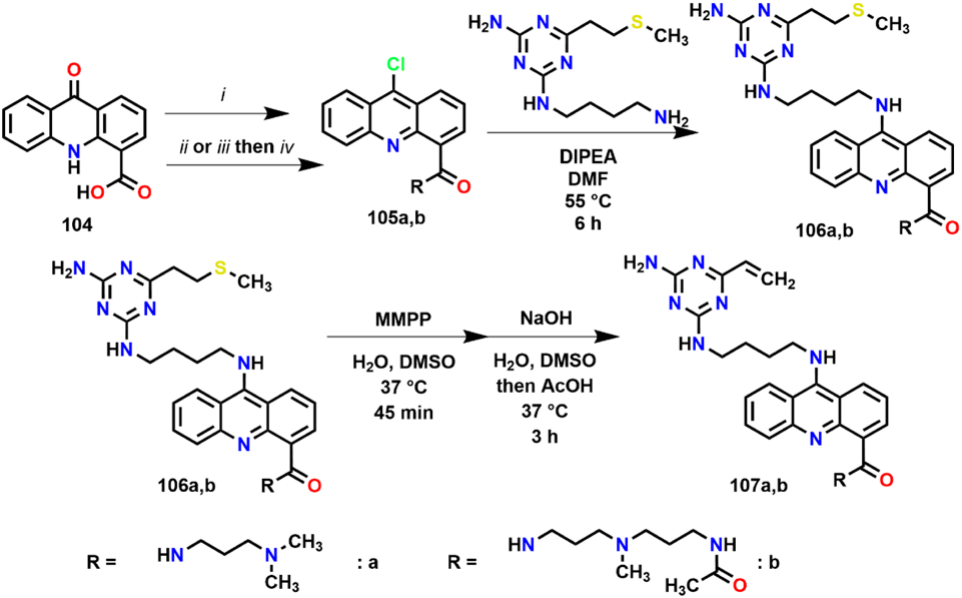
Synthesis of acridine-VDAT conjugates. (i) SOCl_2_, DMF, 70 °C; (ii) *N*,*N*-dimethylpropane-1,3-diamine, DIPEA, DCM, 0 °C to r.t.; (iii) *N*-(3-aminopropyl)-*N*-methylpropane-1,3-diamine, DCM, r.t.; (iv) Ac_2_O, pyridine, r.t.

Compared to the previously mentioned dimers, a vastly different, vinyldiaminotriazine (VDAT) conjugated acridine derivative was reported by Hazemi *et al.* combining the Gq binding capabilities of the acridine ring and VDAT's efficiency as an alkylating agent. Starting compound 105a,b was synthesized from carboxyl acridone using thionyl chloride to introduce chlorine atom in the C-9 position followed by amidation reaction involving aminoalkyl primary amines (Scheme 16). Utilizing a base catalyzed substitution reaction 105a,b was linked to the VDAT derivative to produce the thiomethyl precursor 106a,b in poor yields. The end product 107a,b was achieved following the cleavage of the thiomethyl moiety utilizing magnesium monoperoxyphthalate (MMPP) and subsequent treatment with sodium hydroxide.^53^

## 3 1,10-Phenanthroline derivatives

In the realm of Gq binding molecules, 1,10-phenanthroline derivatives still provide prevalent scaffolds in the pursuit of extending our libraries of potential anticancer agents. To acquire these compounds most common transformations include amidation reactions in the C-2 and C-9 positions and condensation reactions on the B ring to extend the aromatic system. Additional modifications are also carried out on the newly synthesized side chains and aromatic rings – according to the established key principles – to further enhance the biological activity of these analogues.

### 3.1 Symmetric 1,10-phenanthroline amide derivatives

**Scheme 17:**
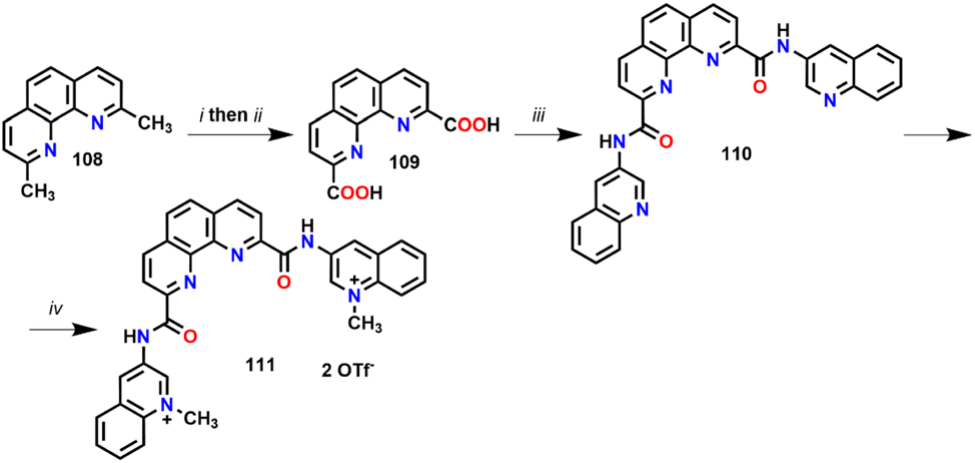
Synthesis of PhenDC3. (i) SeO_2_, 1,4-dioxane, 100 °C, 16 h; (ii) HNO_3_ (70%), 100 °C, 3 h; (iii) EDCI, HOAt, DMF, 23 °C, 16 h; (iv) MeOTf, DCE, 60 °C, 16 h.

One of the first, but certainly the most widely used 1,10-phenathroline Gq binding derivative is PhenDC3. In literature it is regarded as a “gold standard” when it comes to the investigation of potential Gq forming DNA or RNA sequences,^54–56^ as PhenDC3 has exceptional affinity to these genomic structures while also being a synthetically approachable compound. The synthesis of this molecule was first carried out by De Cian *et al.* Using 2,9-dimethyl-1,10-phenanthroline as starting compound the first step of the synthesis includes the oxidation of the alkyl groups using SeO_2_ as oxidizer to carboxylic acid (Scheme 17). 3-Aminoquinoline was then used as the ligand for the amidation reaction which was carried out using EDCI and HOAt as coupling agents.^31^ The final product (111) was achieved through salt formation using triflic acid, thus providing a cationic center in the amide side chain.

**Scheme 18:**
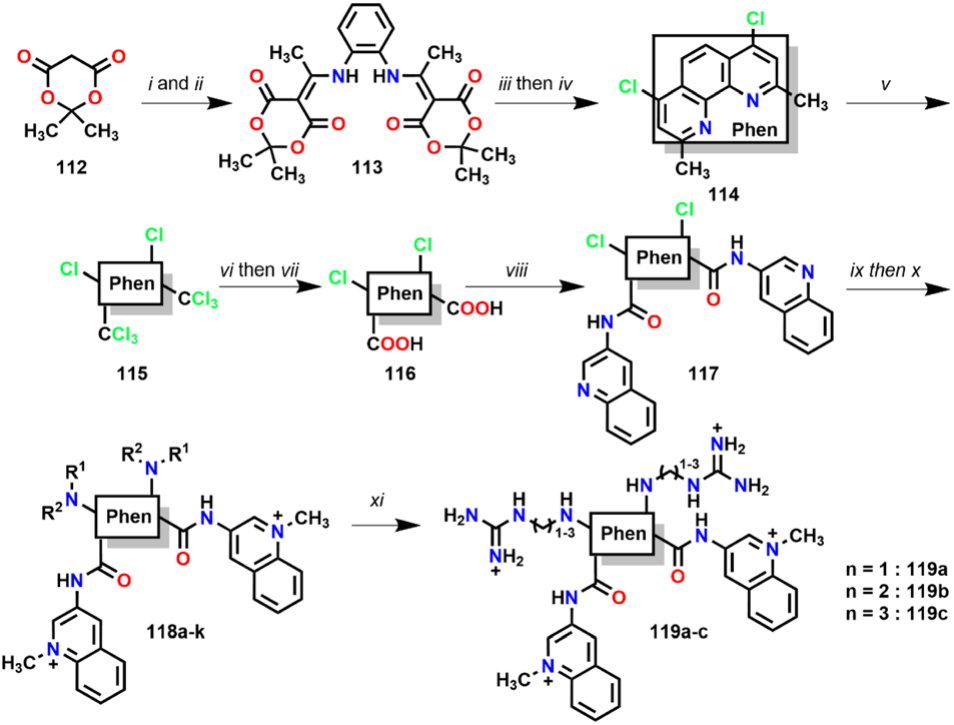
Synthesis of PhenDC3 analogues. (i) Trimethyl-orthoacetate, reflux, 15 min; (ii) 1,2-diaminobenzene, reflux, 2 h, then r.t., 16 h; (iii): Ph_2_O, reflux, 30 min; (iv) POCl_3_, reflux, 3.5 h; (v) NCS, Bz_2_O_2_, CHCl_3_, reflux, 16 h; (vi) H_2_SO_4_, 95 °C, 2 h; (vii) H_2_O, reflux, 1 h; (viii) 3-aminoquinoline, EDC, HOBt, DMF, r.t., 3 h; (ix) DMF, NH_3_ (aq), 100 °C (M.W.), 16 h (in case of compound 118a) or H_2_N(CH_2_)_*n*_NH_2_/HNRR′, 100 °C (M.W.), 20 min, then HCl, H_2_O; (x) CH_3_I, DMF, 115 °C (M.W.), 60–75 min; (xi) (starting from 118f–h): 1-Amidino pyrazole hydrochloride, DMF, diisopropylethylamine, 100 °C (M.W.), 30–90 min, then CH_3_I, DMF, 115 °C (M.W.), 75–90 min.

As a continuation of this work Larsen *et al.* investigated the possibility of further functionalization on the C-4 and C-7 positions. In previous work the research group came to the conclusion that the introduction of amine moieties to these positions also provided effective Gq binders. Based on that it can be enumerated that the 4,7-diamino analogues of the 1,10-phenanthroline-2,9-carboxamides can lead to more biologically active compounds. To synthesize the starting molecule necessary to achieve this, Larsen *et al.* used a reaction involving thermic ring closure reminiscent to the Conrad–Limpach procedure (Scheme 18). The enamine intermediate 113 was obtained through the reaction of Meldrum's acid, trimethyl orthoacetate and 1,2-diaminobenzene. The thermic ring closure and subsequent decarboxylation was carried out at 260 °C using Ph_2_O as solvent. Treatment with POCl_3_ introduced the chlorine atom to the C-4 and C-7 position essential for further transformations. The previously used Se_2_O proved to be ineffective at oxidizing the methyl functions to carboxylic acid, thus NCS promoted radical chlorination was used to achieve reactive trichloromethyl moieties. After hydrolysis with sulfuric acid 116 was obtained in satisfactory yields. The PhenDC3 analogue of 116 was then synthesized using a similar method described by De Cian *et al.*, substituting HOAt with HOBt. The subsequent nucleophilic substitution reactions were carried out in microwave reactor, extending it to primary, secondary amines and even diamines as well (118a–k) (Table 6). In case of the diamine compounds additional introduction of amidine function was possible through the use of 1-amidinopyrazole (119a–c).^57,58^

**Table 6:** Definition of R moieties described in Scheme 18

Conditions	NR^1^R^2^	Compound
115 °C, 60 min	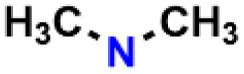	118a
115 °C, 120 min	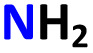	118b
115 °C, 90 min	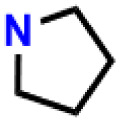	118c
115 °C, 75 min	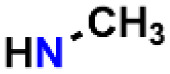	118d
115 °C, 75 min	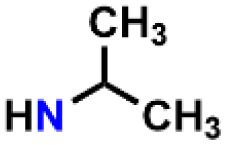	118e
115 °C, 75 min	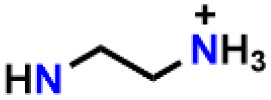	118f
115 °C, 90 min	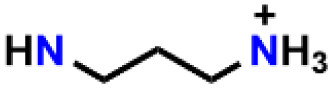	118g
115 °C, 75 min	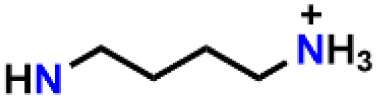	118h
115 °C, 60 min	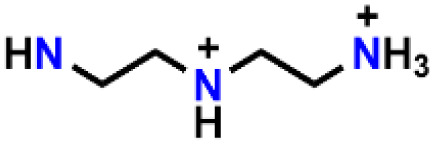	118i
125 °C, 16 h	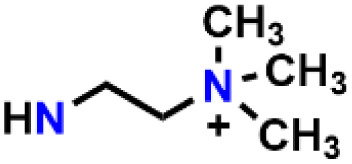	118j
125 °C, 16 h	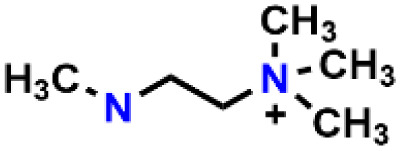	118k

**Scheme 19:**
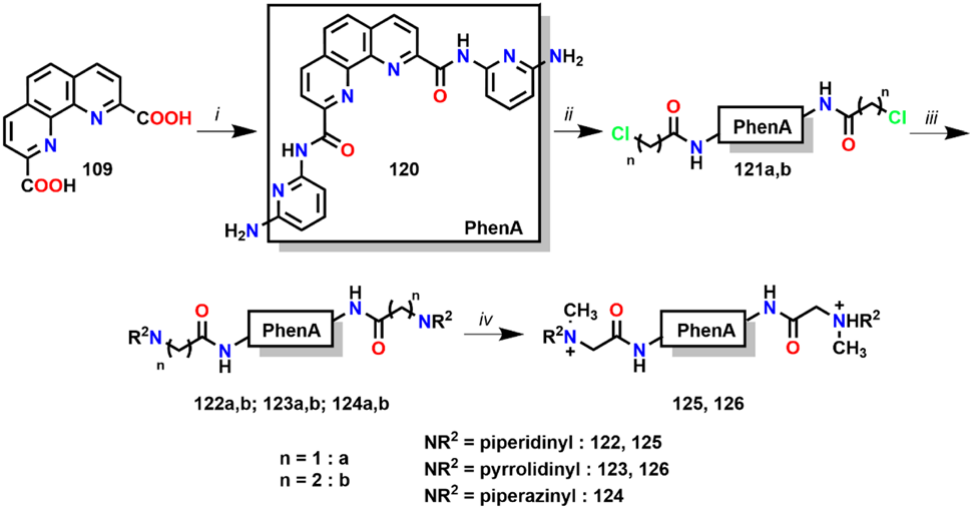
Synthesis of 1,10-phenanthroline pyridine amide derivatives. (i) 2,6-Diaminopyridine, EDCI, HOAt, DMF, 3 h; (ii) chloroacetyl chloride (in case of a) or chloropropanoyl chloride (in case of b), DMF, pyridine, 0 °C to 40 °C, 48 h; (iii) R_2_NH, KI, EtOH, reflux, 3.5 h; (iv) (starting from 122a or 123a) CH_3_I, CHCl_3_, 40 °C, 72 h.

With a switch from quinoline to pyridine, the effect of the amide side chain on Gq affinity was further investigated by Wei *et al.* By using 2,6-diaminopyridine, after the coupling reaction additional functionalization was also made possible (Scheme 19). Furthermore, reacting the second amine moiety of the pyridine function with halogenated acyl halides, followed by a nucleophilic substitution involving a cyclic secondary amine the extension of the amide side chain was carried out (122–124a,b). The compounds with subpar water solubility were then reacted with methyl iodide to form quaternary ammonium salts (125, 126).^59^

**Scheme 20:**
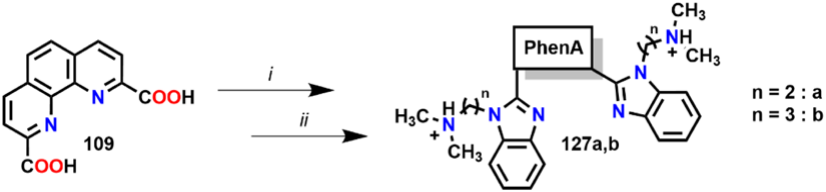
Synthesis of guanine isostere derivatives. (i) 1-(2-(Dimethylamino)ethyl)-1*H*-benzo[*d*]imidazole-2-amine (in case of 127a) or 1-(3-(dimethylamino)propyl)-1*H*-benzo[*d*]imidazole-2-amine (in case of 127b), EDC.HCl, HOBt, *N*-Me-morpholine, DCM, r.t., 24 h; (ii) TFA, DCM, r.t., 24 h.

It has long since been known that Gq loops are for the most part composed of guanines. It can be surmised, that combining a purine isostere molecule with the structural uniqueness of the phenanthroline amides could lead to promising results. This was achieved by Dhamodharan *et al.* using an aminoalkylated analogue of the purine isostere benzimidazole, resulting in 127a,b (Scheme 20). The reactions were carried out utilising common coupling reagents with moderate yields. Dhamodharan *et al.* also investigated pyridine and 1,8-naphtiridine as potential central ring systems however their Gq affinity underperformed compared to 1,10-phenanthroline.^60^

**Scheme 21:**
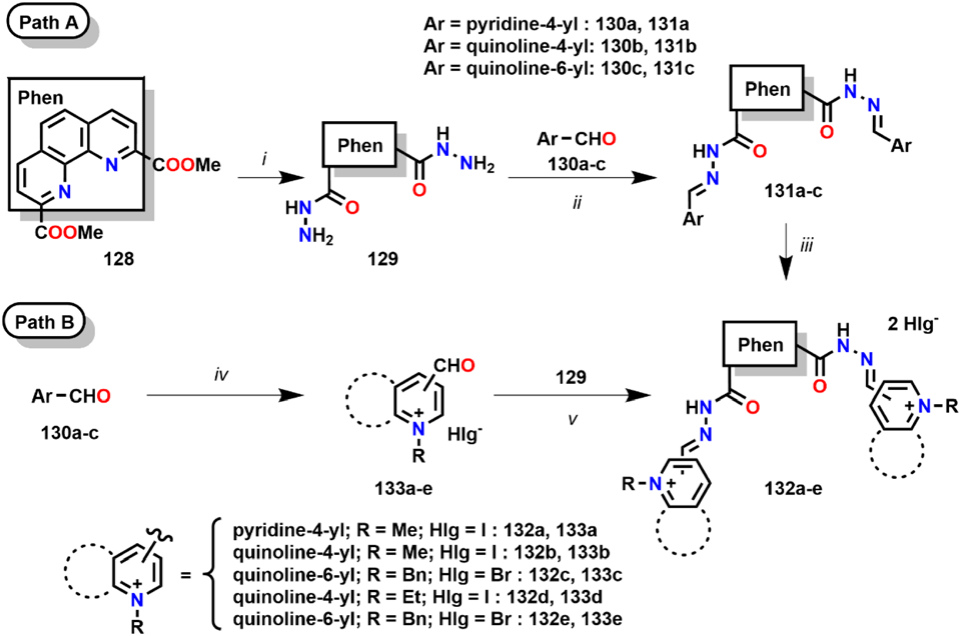
Synthesis of the acylhydrazone derivatives. (i) H_2_N-NH_2_.H_2_O, EtOH, reflux, 18 h; (ii) EtOH, reflux, 18 h; (iii) R-Hlg, DMF, 40 °C or 60 °C, 18 h; (iv) R-Hlg, DCM, r.t., 72 h or acetone, reflux, 18 h; (v) DMF, 80 °C, 2h.

The synthesis of PhenDC3 analogues was further investigated by Reznichenko *et al.* who employed acylhydrazone linkers between the 1,10-phenanthroline ring and the *N*-heteroaromatic scaffolds found in previously synthesized amide derivatives. Two pathways were developed to achieve these compounds (Scheme 21). The shared compounds 129 was synthesized *via* direct amidation using hydrazine hydrate. The hydrazide was then reacted with the corresponding aryl aldehydes (path A) followed by salt formation using alkyl halides to give the end products 132a–c. While this path worked for the majority of derivatives the formation of undesired side products resulted in lower yields overall. This was circumvented by the implementation of path B which involves the synthesis of the alkylated *N*-heterocyclic aldehyde 133a–e before forming the acylhydrazone linker moiety. This resulted sufficiently pure end products in moderate to excellent yields.^61^

**Scheme 22:**
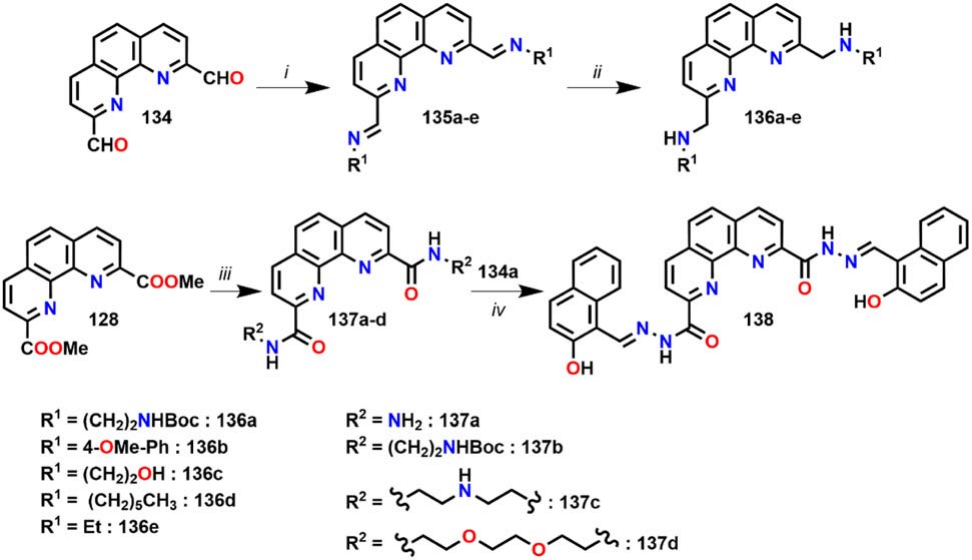
Synthesis of 1,10-phenanthroline derivatives 136a–e, 137a–d and 138. (i) RNH_2_, CHCl_3_/MeOH, reflux, 2–3 h; (ii) NaBH_4_, MeOH, reflux, 2.5–3 h; (iii) RNH_2_ or NH_2_RNH_2_, EtOH (137a) or CHCl_3_/MeOH (137b,c) or CHCl_3_/EtOH (137d), reflux, 3 h (137a) or 24 h (137b,c) or 96 h (137d); (iv) 2-hydroxy-1-naphthaldehyde, H_2_SO_4_, EtOH, reflux, overnight.

Hydrazone and imine derivatives were also synthesized by Figueiredo *et al.* albeit these derivatives were subsequently transformed. Starting from 1,10-phenanthroline aldehyde and reacting it with primary aliphatic and aromatic amines the research group achieved the synthesis of numerous imines, followed by a simple reduction utilizing sodium borohydride to achieve the end products 136a–e (Scheme 22). These compounds due to the presence of cationic centers in their side chain can presumably interact with the negatively charged phosphate groups found in Gq loops. Amide derivatives were also investigated, compound 137a–d were achieved using hydrazine and alkyl diamines as side chains, however these molecules appear to have subpar reactivity reflected in the acquired yields.^62^

**Scheme 23:**
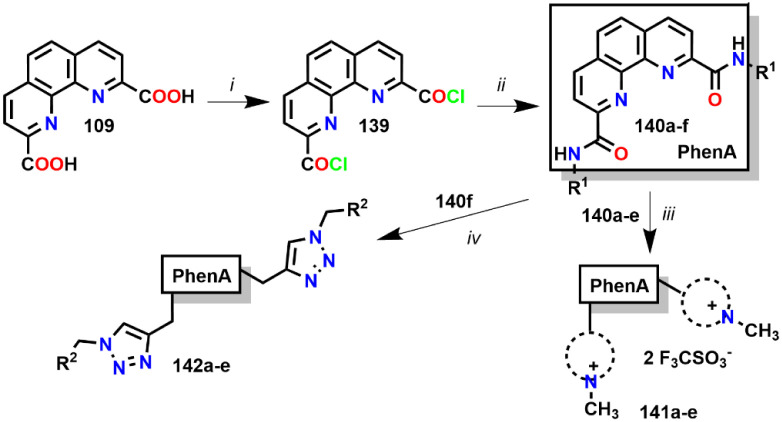
Synthesis of *N*-heterocyclic amide derivatives. (i) (COCl)_2_, DMF, DCM, reflux; (ii) amine/Et_3_N, CHCl_3_ or ACN (anhydr.), r.t.; (iii) CF_3_SO_3_CH_3_, CHCl_3_, reflux; (iv): R^2^CH_2_N_3_, CuSO_4_, Na-ascorbate, *t*-BuOH/H_2_O, 50 °C.

Craciun *et al.* continued to extend the library of PhenDC3 analogues by introducing novel *N*-heterocyclic and thiazole rings into the amide side chain (Scheme 23). The usage of coupling reagents was omitted as the research group instead utilized carboxylic acid chloride 139 to form the amide derivatives 140a–f. Introduction of positive charge into the molecule can further enhance Gq affinity, thus MeOTf was utilized to form the corresponding triflate salts in case of compounds 140a–e, resulting in 141a–e. In the chosen amines, propargyl amine was also included, which opened up the path to further functionalization as the formation of triazole rings in the side chain could be achieved *via* the “click” cycloaddition reaction. This led the way to further branching amides (142a–e) that can be beneficial regarding Gq affinity (Table 7).^63^

**Table 7:** Definition of R functions described in Scheme 23

R^1^	R^2^	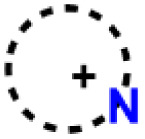	Compound
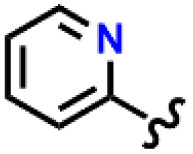	n.a.	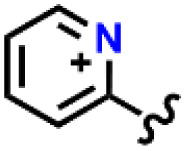	140a; 141a
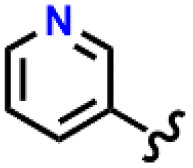	n.a.	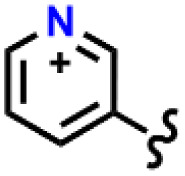	140b; 141b
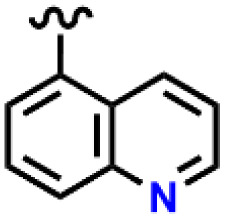	n.a.	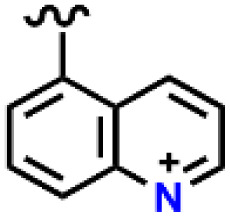	140c; 141c
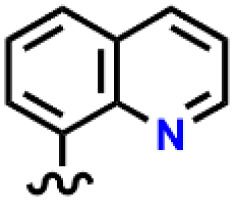	n.a.	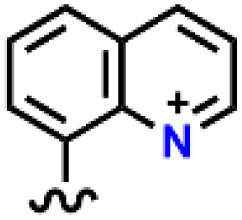	140d; 141d
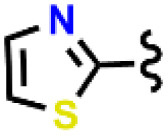	n.a.	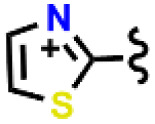	140e; 141e
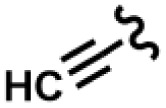	n.a.	n.a.	140f
n.a.	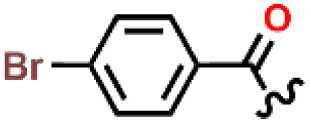	n.a.	142a
n.a.	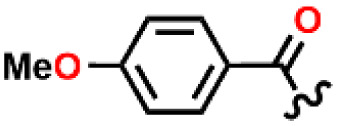	n.a.	142b
n.a.	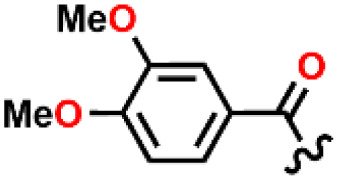	n.a.	142c
n.a.	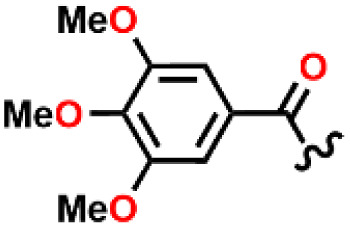	n.a.	142d
n.a.	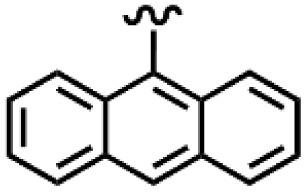	n.a.	142e

### 3.2 Symmetric non-amide derivatives

**Scheme 24:**
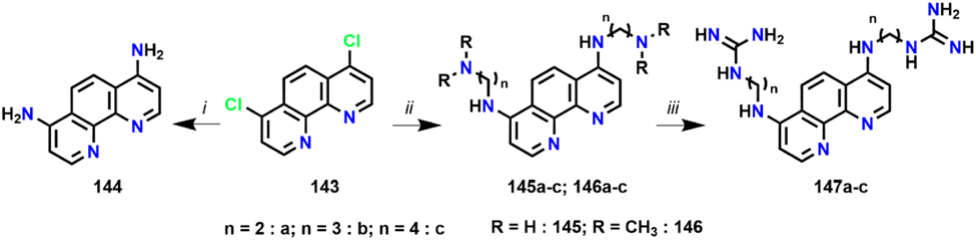
Synthesis of C-4 and C-7 substituted derivatives. (i) cc.NH_3_, 120 °C (M.W.), 1 h; (ii) RNH_2_, EtOH, 165 °C (M.W.), 16 h; (iii) 145a–c, 1*H*-pyrazole-1-carboxamidine hydrochloride, DIPEA, DMF, 60 °C, overnight.

The amide moiety in the C-2 and C-9 position, while contributing significantly to the Gq affinity of the molecule is not entirely a necessity. Gq binding compounds not bearing the aforementioned function were investigated by several research groups albeit the symmetrical component was kept thus sharing similar qualities to the previously defined molecules. Among the first of these publications Nielsen *et al.* described the synthesis of several diamino and aminoalkylated diamino compounds with a key difference being C-4 and C-7 substitutions instead of C-2 and C-9 (Scheme 24). Starting from 4,7-dichloro-1,10-phenanthroline and following a microwave assisted nucleophilic substitution using excess primary amines or ammonia 144 and 145a–c and 146a–c were achieved with relatively high yields. In the case of derivatives bearing unreacted primary amine moieties (145a–c) the side chain was treated with 1*H*-pyrazole-1-carboxamidine to obtain their guanidine analogues (147a–c) thus introducing further cationic centers to the compounds.^64^

**Scheme 25:**
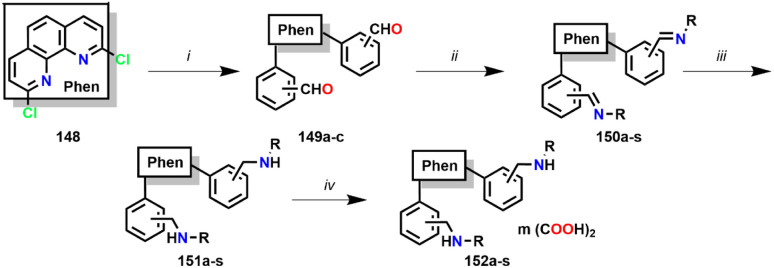
Synthesis of derivatives achieved utilizing Suzuki–Miyaura reaction. (i) (4-formylphenyl)boronic acid, Pd(PPh_3_)_4_, K_2_CO_3_, toluene, reflux; (ii) RNH_2_, EtOH/reflux or toluene/molecular sieves/r.t., 24 h; (iii) NaBH_4_, MeOH, r.t., 2 h; (iv) (COOH)_2_, IPA, reflux, 30 min.

However, the C-2 and C-9 atoms are still distinguished regarding investigative effort as the majority of literature is still involved in the modification of these positions. Several articles reported the synthesis of a wide variety of molecules further reinforcing the importance of these positions. Compounds 149a–c were prepared using a double Suzuki–Miyaura cross coupling reaction starting from 2,9-dichloro-1,10-phenathroline with formylphenylboronic acid regioisomers (Scheme 25). This reaction was catalyzed by Pd(PPh_3_)_4_ and alkaline carbonates were used as bases. Schiff-bases 150a–s were achieved by the reaction of 149a–c and different aminoalkyl and *N*-heteroaromatic alkyl primary amines which was followed by their reduction into secondary amines utilizing sodium borohydride, resulting in 151a–s. Using the same procedure other notable compounds were synthesized including 1,10-phenanthroline dimers and trimers linked through a branching secondary and tertiary amine derivatives.^65^

**Scheme 26:**
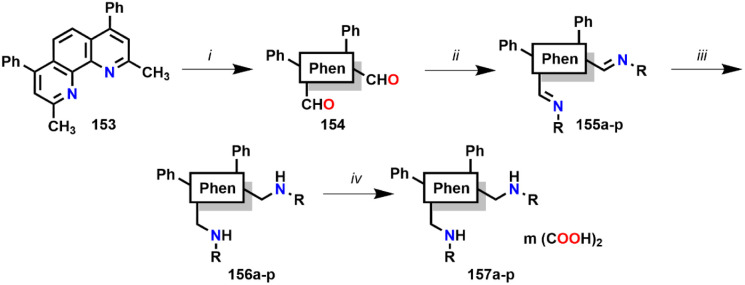
Synthesis of bathocuproine derivatives. (i) SeO_2_, 1,4-dioxane, reflux, (ii) (4-formylphenyl)boronic acid, Pd(PPh_3_)_4_, K_2_CO_3_, toluene, reflux; (iii) RNH_2_, EtOH/reflux or toluene/molecular sieves/r.t., 24 h; (iv) NaBH_4_, MeOH, r.t., 2 h; (iv) (COOH)_2_, IPA, reflux, 30 min.

This work was extended to 4,7-phenyl substituted derivatives with a key difference being the omission of the linking phenyl ring between 1,10-phenanthroline and the formyl moiety (Scheme 26). This necessitated the application of an additional step – the oxidation of the methyl groups of the starting material bathocuproine (153) to aldehydes (154) using selenium dioxide. To achieve the desired compounds 156a–p starting from 154 the same methods were utilized described previously by Gueddouda *et al.* with the only difference being a solvent change during the formation of the Schiff-base. It is interesting to mention that despite the differences between the used 1,10-phenanthroline derivatives similar yields were achieved during the syntheses (Tables 8 and 9).^66–68^

**Table 8:** Definition of R functions described in Scheme 25

R	Pos.	Comp.	R	Pos.	Comp.
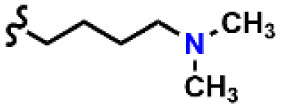	4	a	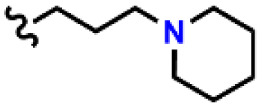	4	k
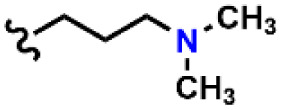	4	b	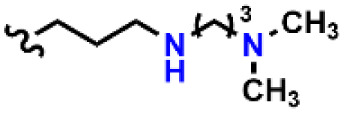	4	l
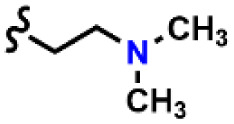	4	c	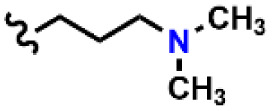	3	m
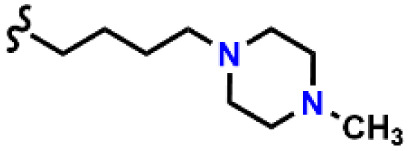	4	d	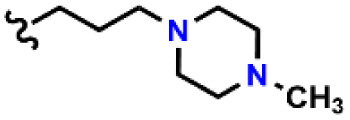	3	n
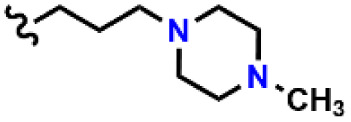	4	e	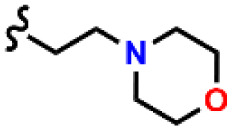	3	o
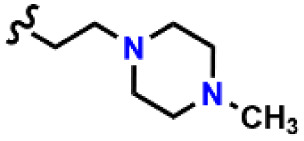	4	f	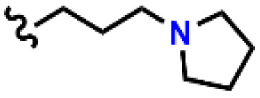	3	p
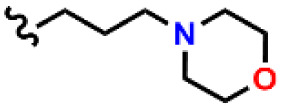	4	g	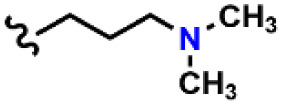	2	q
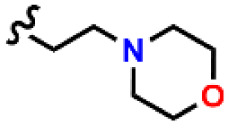	4	h	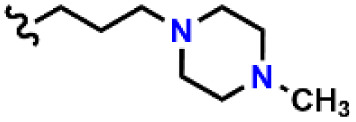	2	r
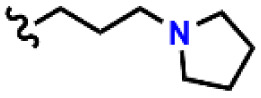	4	i	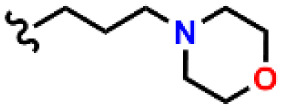	2	s
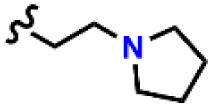	4	j			

**Table 9:** Definition of R functions described in Scheme 26

R	Compound	R	Compound
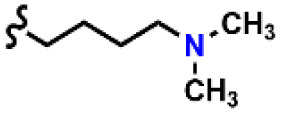	a	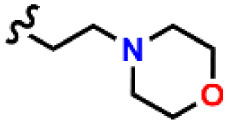	i
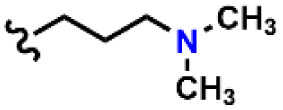	b	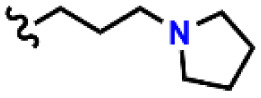	j
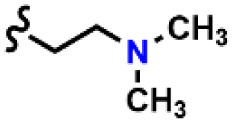	c	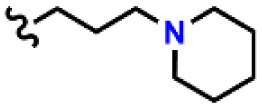	k
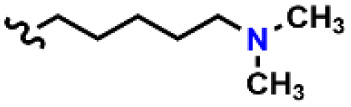	d	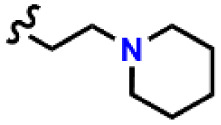	l
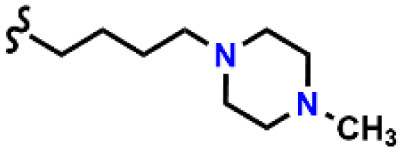	e	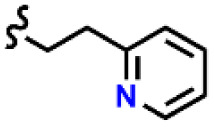	m
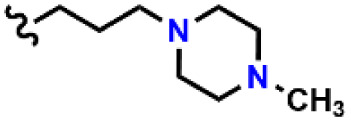	f	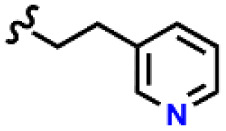	n
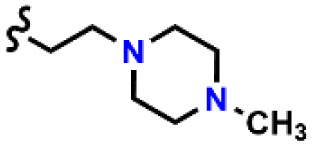	g	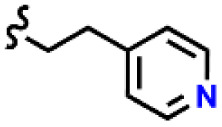	o
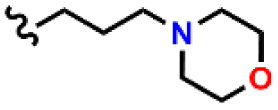	h	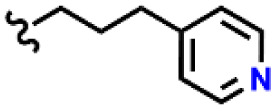	p

**Scheme 27:**
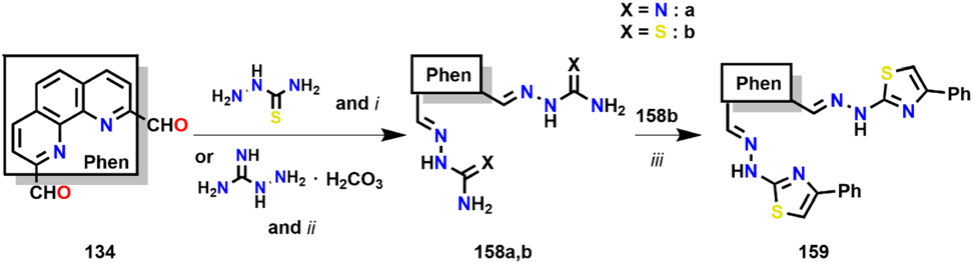
Synthesis of 1,10-phenanthroline imides. (i) MeOH, H_2_SO_4_, 100 °C, 1 h; (ii) HCl, reflux, 1 h then r.t.; KOH, reflux, 10 min; (iii) 2-bromoacetophenone, EtOH, 100 °C, 24 h.

In the following years, using guanidine derivatives additional 1,10-phenanthroline imides were synthesized. To achieve the synthesis of the planned compounds, aminoguanidine bicarbonate and thiosemicarbazide were reacted with the phenanthroline dialdehyde in basic or acidic conditions respectively (Scheme 27). The thioamide derivative was further reacted with 2-bromoacetophenone *via* a Hantzsch-type cyclodehydration reaction to form the phenylthiazole function in the side chain. Both 158a (codenamed PhenQE8) and 159 was obtained with 68% and 75% yields respectively with *E* as the preferred isomer.^69,70^

**Scheme 28:**
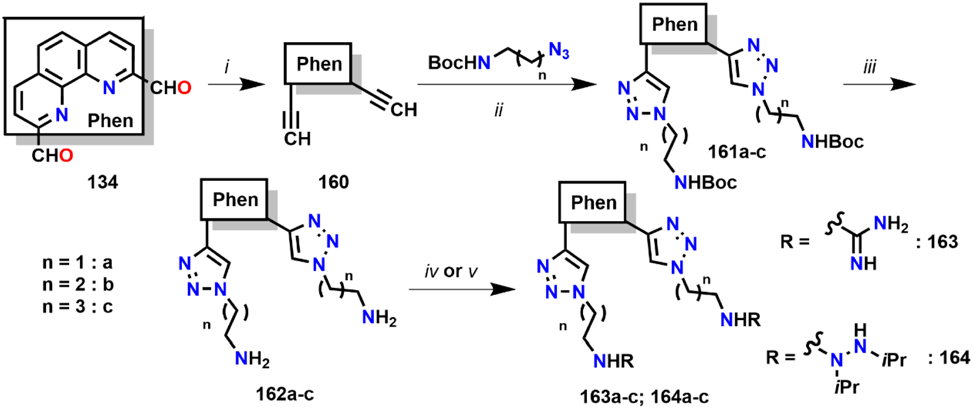
Synthesis of triazole derivatives 163a–c and 164a–c. (i) Dimethyl-1-diazo-2-oxopropylphosphonate (Ohira–Bestmann reagent), K_2_CO_3_, MeOH, 3 h; (ii) Cu(ii)-TBTA complex, Na-ascorbate, DCM/H_2_O, 24 h, r.t.; (iii) TFA, DCM, 2 h, r.t.; (iv) (in case of 163a–c): 1*H*-pyrazole-1-carboxamidine hydrochloride, TEA, EtOH, 120 °C (M.W.), 30 min; (v) (in case of 164a–c): DIC, TEA, EtOH, 120 °C (M.W.), 30 min.

**Scheme 29:**
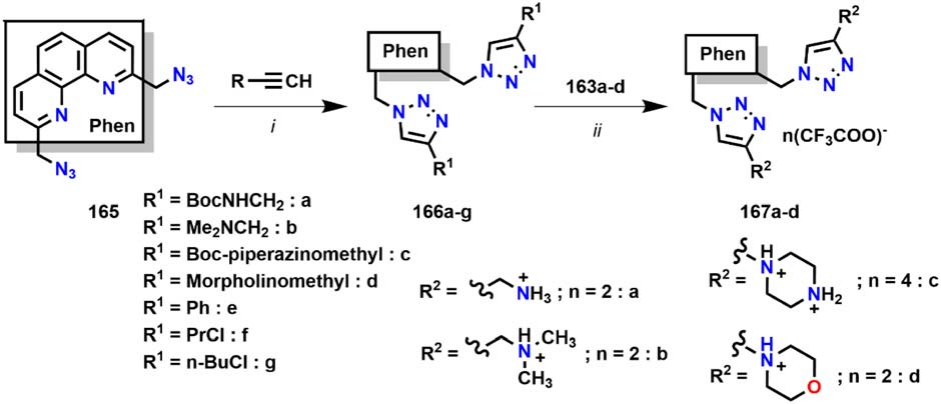
Synthesis of triazole derivatives 166a–g and 167a–d. (i) Na-l-ascorbate, Cu(ii)-TBTA complex (6 mol%), DIPEA, DCM/H_2_O, r.t., 24–96 h; (ii) TFA, DCM, r.t. or TFA, r.t.

To achieve five membered rings the popular “click” reaction was also employed. However, the approach to achieve these compounds was different throughout the years. Nielsen *et al.* utilized Ohira–Bestmann reagent to form the necessary alkyne function on the 1,10-phenanthroline ring (Scheme 28), while Figueiredo *et al.* envisioned the usage of diazidomethyl 1,10-phenathroline as the starting compound (Scheme 29). The triazole ring incorporating intermediates 161a–c were achieved through the reaction of protected aminoalkyl azides with 160 in the presence of sodium-ascorbate and copper(ii). As the 1,10-phenanthroline ring has exceptional complex forming capabilities the tris(benzyltriazolylmethyl)amine complex of Cu(ii)-TBTA (Tris(benzyltriazolylmethyl)amine) was used to improve yields (Scheme 29). After removal of the Boc protecting-group gave 162a–c, further functionalization was achieved utilizing pyrazole-1-carboxamide or DIC on the primary amine moiety, resulting in 163a–c and 164a–c.^71^

Regarding the 1,10-phenathroline diazide, the triazole derivatives 166a–g were formed through similar methods as utilized by Nielsen *et al.* with the addition of DIPEA as base and using aminoalkyl, chloroalkyl, aryl and cyclic secondary amine substituted alkynes (Scheme 29). The trifluoro acetate salt was also obtained in the case of derivatives containing amine moiety in the triazole side chain (167a–d).^72^

**Scheme 30:**
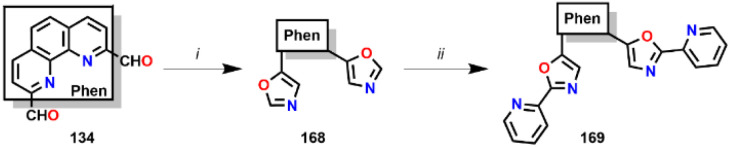
Synthesis of 1,10-phenanthroline oxazole derivatives. (i) TosMIC, K_2_CO_3_, MeOH, 10–20 °C, 2 h, then reflux, 4 h; (ii) P(cyclohexyl)_3_HBF_4_, Pd(OAc)_3_, CuI, 2-Br-pyridine, Cs_2_CO_3_, 1,4-dioxane, 130 °C, 24 h.

The circle of five membered ring derivatives was closed by Medeiros-Silva *et al.* with the inclusion of oxazole in the side chain. To achieve this the Van Leusen reaction was employed.^73^ The synthesis includes the usage of TosMIC (toluenesulfonylmethyl isocyanide) to form the oxazole ring through cyclization and subsequent elimination to obtain 168 (Scheme 30). For the reaction to take place, a strict temperature control was necessary for the oxazole formation, meaning 10–20 °C had to be maintained while reflux temperature was employed for the tosyl elimination. After a cross-coupling reaction involving 2-bromopyridine and tricyclohexylphosphine tetrafluoroborate, copper iodide, palladium(ii)-acetate as catalysts 169 was achieved in low yields.^74^

**Scheme 31:**
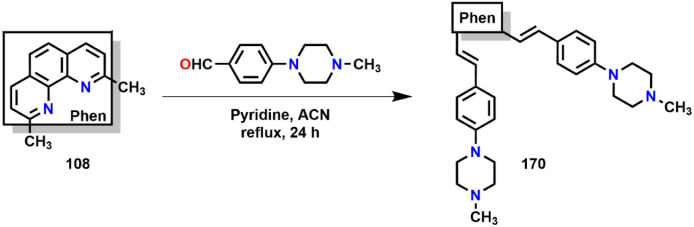
Synthesis of the styryl linked 1,10-phenanthroline derivatives.

Based on a previously reported carbazole linked bis-benzimidazole derivative^75^ Wu *et al.* described the synthesis of styryl linked 1,10-phenanthroline compounds (Scheme 31). Following a simple procedure involving 2,9-dimethyl-1,10-phenanthroline and 4-(4-methylpiperazin-1-yl) benzaldehyde refluxed at basic conditions 170 was obtained; however, the authors did not describe yields for them, making the procedure unreliable.^76^

### 3.3 Asymmetric 1,10-phenanthroline derivatives

**Scheme 32:**
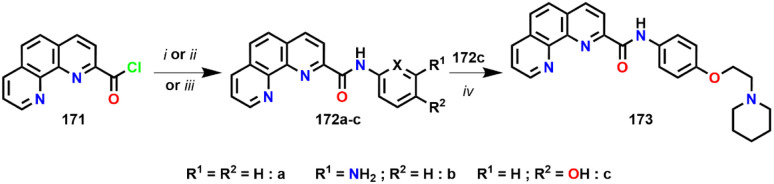
Synthesis of 1,10-phenanthroline derivatives 172a–c and 173. (i) 2-Aminopyridine, TEA, DCM, r.t., overnight (to achieve 172a); (ii) 2,6-diaminopyridine, TEA, THF/DCM, reflux (to achieve 172b), 8 h; (iii) 4-((trimethylsilyl)oxy)aniline, TEA (to achieve 172c); (iv) 1-(2-chloroethyl)piperidine, NaH, DMF, 110 °C.

While the symmetrical component of 1,10-phenanthroline is beneficial in terms of Gq affinity based on previous findings mentioned in the last two chapters, its presence could be omitted while retaining competent Gq binding capabilities. To reinforce this observation Reed *et al.* reported the synthesis of C-2 amide derivatives (172a–c) and their Pt(II) complexes (Scheme 32). To achieve these compounds, the carboxylic acid chloride of 1,10-phenanthroline (171) was reacted with aniline derivatives (in the case of 172c*O*-protected aniline derivative), while the *p*-anisidine analogue further functionalized using 1-(2-chloroethyl)piperidine, resulting in 173. The investigated complexes were obtained using K_2_PtCl_4_ as Pt(II) source for a central atom, resulting in acceptable to poor yields.^77,78^

**Scheme 33:**
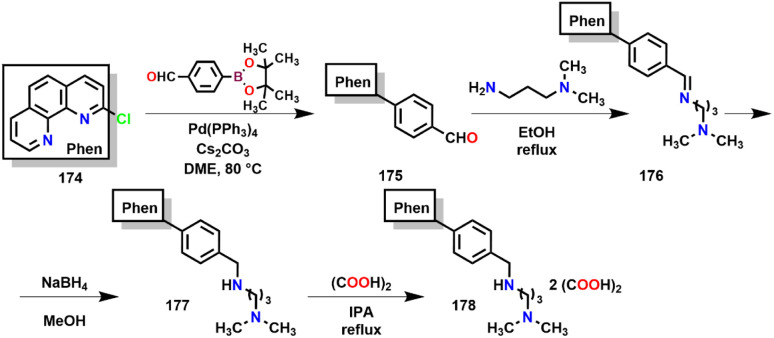
Synthesis of asymmetric 1,10-phenanthroline derivative 178.

**Scheme 34:**
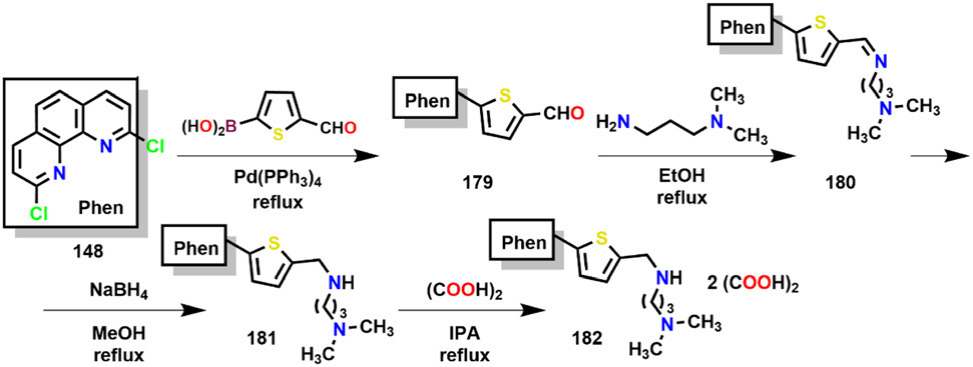
Synthesis of asymmetric 1,10-phenanthroline derivative 182.

Previously mentioned, but also relevant here are the research projects spearheaded by Gueddouda *et al.* and Guillon *et al.* The synthesis of symmetrical 1,10-phenanthroline derivatives was the main focus of these investigations, however using similar methods as described above asymmetrical compounds were achieved as well (178) (Scheme 33). Compound 179 is worthy of further mention as a symmetrical derivative containing the thiophene ring in the side chain could not be achieved, only the asymmetrical analogue 182 was reported by the research groups (Scheme 34).^65,66^

**Scheme 35:**
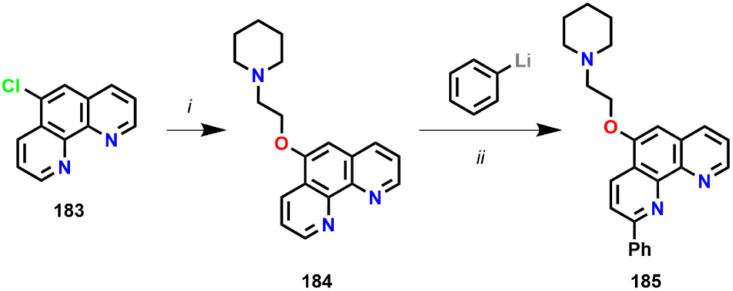
Synthesis of piperidinoethyl derivatives. (i) 1-(2-Hydroxyethyl)piperidine, KOH, DMSO, 60 °C, 4 h; (ii) toluene, 0 °C, 3 h, then MeOH, MnO_2_, 24 h.

Featuring a similar piperidinoethyl function as derivatives reported by Reed *et al.* 1,10-phenanthroline analogues substituted in the C-2 and C-5 positions were described by Suntharalingam *et al.* Phenyl analogues of 1,10-phenanthroline can act as a tridentate ligand while also being potent Gq binding compounds as reported in literature. However, their Gq selectivity over duplex DNA are poor, thus the piperidinoethyl function was introduced in the C-5 position to improve this property while also enhancing the water solubility of the molecule (Scheme 35). Starting from 5-chloro-1,10-phenanthroline and using 1-(2-hydroxyethyl)piperidine in a base catalyzed nucleophilic substitution reaction 184 was synthesized with satisfactory yields. The C-2 functionalization was accomplished using phenyllithium, resulting in relatively high yields.^79^ The Pt(II) complex of 185 was achieved in a similar fashion described by Reed *et al.*^78^ Other C-5 substituted derivatives of 1,10-phenanthroline were synthesized by Singh *et al.* this time with the introduction of neomycin to improve Gq affinity.^80^

**Scheme 36:**
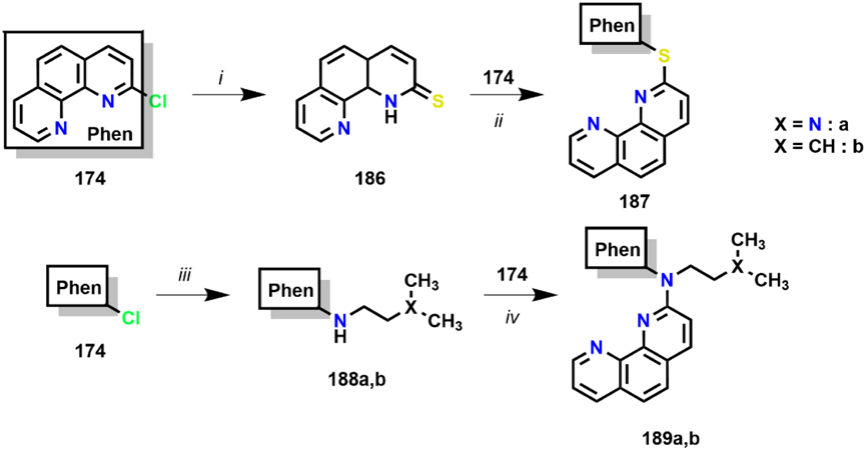
Synthesis of *S* and *N* linked bis-1,10-phenanthrolines. (i) NaSH or Na_2_S, DMF; (ii) Ph_2_O, reflux, then KOH/H_2_O; (iii) *N*,*N*-dimethyl ethylenediamine (to 188a) or isoamyl amine (to 188b), reflux; (iv) NaH, DMF, reflux.

**Scheme 37:**
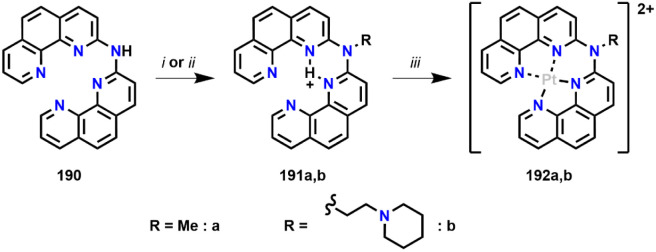
Synthesis of bis-1,10-phenanthroline Pt complexes. (i) CH_3_I, DMSO, KOH, r.t., 30 min (to achieve 191a); (ii) KOH, DMSO, r.t., 2 h, then 1-(2-chloroethyl)piperidine hydrochloride, 110 °C, 8 h (to achieve 191b); (iii) Pt(dmso)_2_Cl_2_, MeOH/H_2_O, reflux, 5 h.

Considering the nature of symmetrical qualities compounds mentioned in the previous chapter possess, the following molecules are discussed as asymmetric derivatives. Bianco *et al.* and He *et al.* reported the synthesis of *S*- and *N*-linked bis-1,10-phenanthroline derivatives with the latter being further functionalized using alkyl and aminoalkyl substituents (Schemes 36 and 37). To achieve compound 187, 2-chloro-1,10-phenanthroline (174) was reacted with NaSH or Na_2_S to obtain the 2-thione derivative of 1,10-phenanthroline (186) which was refluxed with 174 in Ph_2_O and treated after with KOH. In the case of the *N*-linked compounds 174 was refluxed with the corresponding primary amine followed by the treatment with a strong base (NaH) and 188a,b yielded 189a,b as the final product (Scheme 36).^81^ He *et al.* applied a different approach as an already linked bis-phenanthroline was reacted with CH_3_I or 1-(2-chloroethyl)piperidine in basic conditions to achieve 191a,b (Scheme 37).

Metal complexes also show the advantages provided by the 1,10-phenanthroline ring; thus numerous compounds were described in literature using a 1,10-phenanthroline derivative as the ligand and Pt(II), Ir(iii), Cu(ii) and Ru(ii) as the central cations.^82–86^ In the case of 191a,b a Pt(dmso)_2_Cl_2_ complex was utilized at reflux temperatures to achieve 192a,b, thus enhancing the Gq affinity of the bis-1,10-phenanthroline derivatives.^87^

**Scheme 38:**
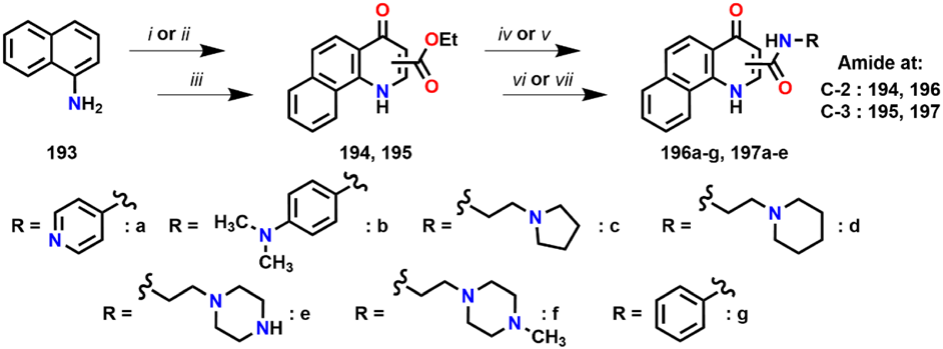
Synthesis of benzo[*h*]quinoline derivatives. (i) Diethyl ethoxymalonate (for 194); (ii) DEAD, MeOH (anhydr.), reflux (for 195); (iii) Ph_2_O, reflux; (iv) NaOH, MeOH, reflux, 2 h; (v) NaOH, dil. HCl; (vi) RNH_2_, EDCI, DMAP, DMF, DCM; (vii) RNH_2_, EDCI, DMAP, DMF.

**Scheme 39:**
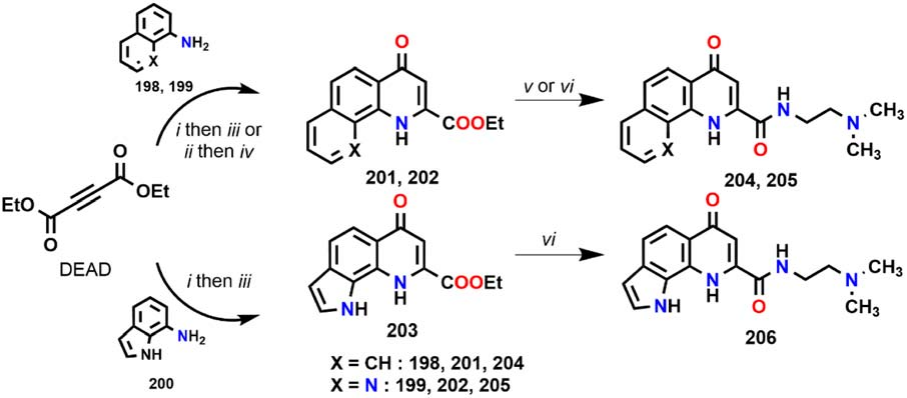
Synthesis of tricyclic KYNA derivatives. (i) DEAD, EtOH, reflux (for 201) or 1,2-dichlorobenzene, r.t. (for 202), 2 h; (ii) DEAD, 1,2-dichlorobenzene, r.t. (for 203) 30 min; (iii) 1,2-dichlorobenzene, reflux, 12 h (for 201) or 24 h (for 203); (iv) 1,2-dichlorobenzene, M.W., 200 °C, 2 h; (v) *N*,*N*-dimethylethylenediamine, EtOH, 120 °C, M.W., 2 h (for 204); (vi) *N*,*N*-dimethylethylenediamine, neat, r.t., 2 h (for 205, 206).

Certain modifications of the 1,10-phenanthroline core can be accomplished without hindering its ability to bind Gq structures, a notable one being the exclusion of the *N* atom in the C-ring thus achieving the benzo[*h*]quinoline scaffold. Paritala *et al.* was among the first to investigate the Gq affinity of said derivatives synthesizing C-2 and C-3 substituted aminoalkyl and aryl amide derivatives (Scheme 38). The synthesis of these compounds was achieved *via* thermic cyclisation of the enamine of 1-naphthylamine using diethyl ethoxy-malonate in the case of 194 and diethyl acetylenedicarboxilate (DEAD) in the case of 195 (better known as the Conrad–Limpach procedure). The amide side chain was formed *via* a coupling reaction of the carboxylic acid of 194 and 195 and various aminoalkyl and aryl primary amines (196a–g and 197a–e).^88^ It should be mentioned that some of the reaction conditions weren't included in the publication hence their omission from the scheme. Using a modified version of the Conrad–Limpach procedure, similar tricyclic analogues can be achieved as well. By substituting the Ph_2_O used in the original procedure with 1,2-dichlorobenzene more facile work-up is possible thus improving yields. Using this alternative method the synthesis of not only the benzo[*h*]quinoline and the 1,10-phenanhtroline but a novel 1*H*-indolo[*h*]quinoline ring (201–203) was achieved starting from 1-naphtylamine, 8-aminoquinoline or 7-amino-1*H*-indole (Scheme 39).^89^

**Scheme 40:**
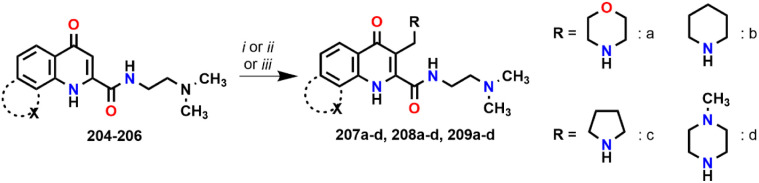
Synthesis of Mannich-bases from tricyclic KYNA derivatives. (i) 204, R_2_NH, CH_2_O, 1,4-Dioxane, reflux, 8 h (for 207a–d); (ii) 205, R_2_NH, CH_2_O, THF, 110 °C, M.W., 4 h (for 208a–d); (iii) 206, R_2_NH, CH_2_O, AcOH, 1,4-dioxane, reflux, 2 h (for 209a–d).

Previous research regarding kynurenic acid (KYNA) and its derivatives showed that the inclusion of tertiary amine moieties in the amide side chain can lead to improved biological activity. Said function can also be formed *via* the modified Mannich-reaction (*m*Mr) using secondary amines as reaction partners.^90,91^ The *m*Mr wasn't previously utilized in literature to functionalize the 1,10-phenanthroline ring to achieve Gq binding molecules, thus its inclusion is certainly an intriguing possibility. Using this aminoalkylation reaction on the aforementioned ring systems and their amide derivatives (Scheme 40), numerous Mannich-bases were achieved *via* previously optimized (in case of 207a–d)^90^ or modified versions of these methods (in case of 208a–d, 209a–d).^89^

### 3.4 Fused 1,10-phenanthroline derivatives

**Scheme 41:**
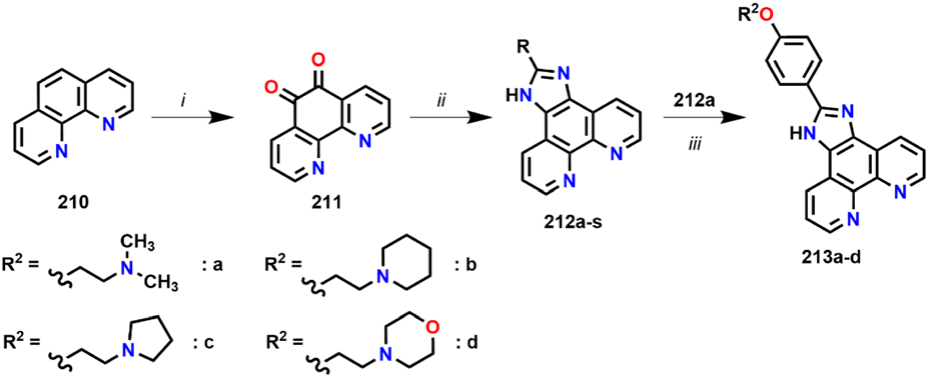
Synthesis of imidazole fused 1,10-phenanthroline derivatives. (i) KBr, H_2_SO_4_/HNO_3_; (ii) R-CHO, NH_4_OAc, AcOH; (iii) R^2^-Cl, NaH, DMF.

One of the more widely discussed transformations performed on the 1,10-phenanthroline ring system are its' fusions with different aromatic rings. The inclusion of a condensed imidazole ring on the B-ring of 1,10-phenanthroline is reported to have a beneficial effect on Gq affinity thus research on these derivatives was enlivened in the mid-2010s. Different analogues (212a–s) were synthesized by numerous research groups using a similar method originally described by Steck and Day in the ‘40 s.^92^ Starting from 1,10-phenanthroline-5,6-dione (211) the reaction was carried out using substituted benzaldehydes, ammonium-acetate and acetic acid as solvent at reflux temperatures (Scheme 41). Slight modifications were made to this scheme to improve reaction times, most notably the inclusion of a microwave-assisted heat transfer, resulting in under half an hour reaction times (Table 10, Entries #5–7). The yields weren't enhanced by these improvements, but this wasn't necessitated, as the original yields were already satisfactory (Table 10, Entries #1–4 *vs.* #5–7). Judging by the acquired yields, different benzaldehyde derivatives had similar behavior, the only outlier being the 4-sulfoxylbenzene derivative (Table 10, Entry #7); however, the cause of the discrepant yield wasn't further elaborated by the research groups.^93–101^ Using 4-hydroxybenzaldehyde, further functionalization was made possible by Wei *et al.* with different aminoalkyl side-chains, thus achieving 213a–d.^102^

**Table 10:** Conditions and acquired yields of imidazole fused 1,10-phenanthroline derivatives

Entry	Temp (°C)	Time (h)	Yield (%)	R
#1 (ref. 100)	Reflux	2	84	4-OH-Ph (212a)
#2 (ref. 91)	Reflux	4	85	Thiophene (212b)
#3 (ref. 92)	Reflux	1	80–90	4-OMe-Ph (212c)
#4 (ref. 93)	Reflux	2	83	Indole (212d)
#5 (ref. 94)	100 (M.W.)	20 min	93.1	4-F-Ph (212e)
			91.7	4-Cl-Ph (212f)
			92.3	4-Br-Ph (212g)
			94.2	2,3-Cl-Ph (212h)
			88.5	3,4-Cl-Ph (212i)
			Unknown	2,4-Cl-Ph (212j)
#6 (ref. 95)	100 (M.W.)	20 min	91.3	3-NO_2_-Ph (212k)
			82.3	3-CF_3_-Ph (212l)
			94.7	3-Cl-Ph (212m)
			89.7	3-OH-Ph (212n)
#7 (ref. 96 and 97)	100 (M.W.)	20 min	85.6	Ph (212o)
			87	2-Sulfoxyl-Ph (212p)
			51.4	4-Sulfoxyl-Ph (212q)
			81.7	2-Br-Ph (212r)
			89.0	4-Br-Ph (212s)

**Scheme 42:**
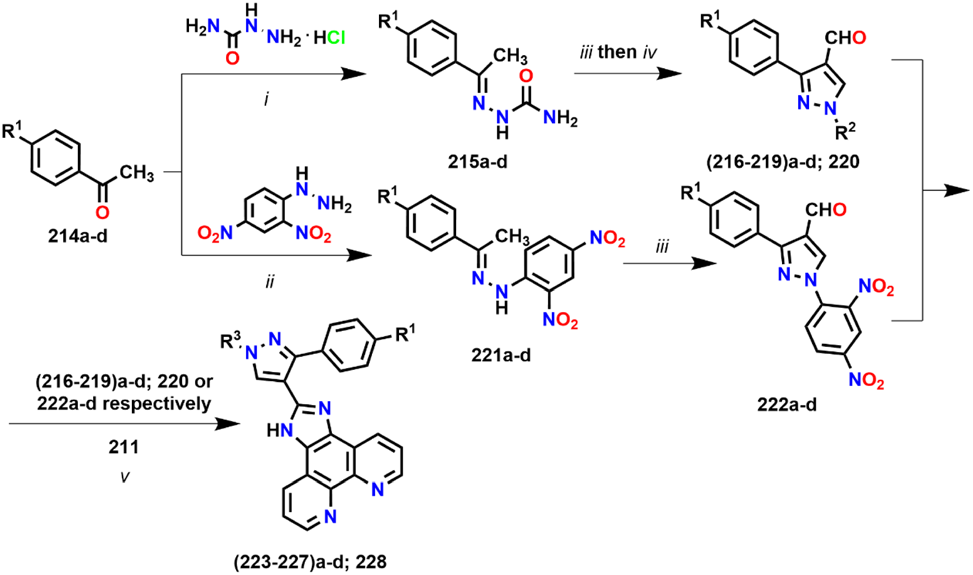
Synthesis of imidazole fused derivatives 223–227a–d and 228. (i) NaOAc, EtOH, 75 °C, 6 h; (ii) EtOH, 75 °C, 6 h; (iii) POCl_3_, DMF, 0–65 °C; (iv) 216a–d, R_2_Cl or CH_3_I (for 217a–d), KI, inorg. base, DMF, 50–80 °C, 6–8 h; (v) NH_4_OAc, AcOH, 90–100 °C, 6 h.

The method described above was also utilized by Liu *et al.*; however, substituted pyrazole aldehydes were chosen by the research group as the focus of investigation (Scheme 42). These functions were prepared individually from substituted acetophenones 214a–d*via* hydrazide formation (215a–d or 221a–d), followed by a ring closure with the use of phosphoryl chloride. In case of 216a–d, further reaction with methyl-iodide (to achieve 217a–d) or benzyl- and aminoalkyl-chlorides (to achieve 218a–d, 219a–d, and 220) resulted in their corresponding derivatives. These aldehydes were reacted with 1,10-phenanthroline-5,6-dione (211) and ammonium acetate in acetic acid at reflux temperature to acquire the final products ((223–227a–d) and 228). Yields were reported at 60–90% intervals, further reinforcing the efficiency of this method (Table 11).^103^

**Table 11:** Definition of R functions described in Scheme 42

R^1^	R^2^	R^3^	Compound	R^1^	R^2^	R^3^	Compound
F	H	H	216a, 223a	Me	Bn	Bn	218d, 225d
Cl	H	H	216b, 223b	F	Me_2_N(CH_2_)_3_	Me_2_N(CH_2_)_3_	219a, 226a
Br	H	H	216c, 223c	Cl	Me_2_N(CH_2_)_3_	Me_2_N(CH_2_)_3_	219b, 226b
Me	H	H	216d, 223d	Br	Me_2_N(CH_2_)_3_	Me_2_N(CH_2_)_3_	219c, 226c
F	Me	Me	217a, 224a	Me	Me_2_N(CH_2_)_3_	Me_2_N(CH_2_)_3_	219d, 226d
Cl	Me	Me	217b, 224b	Br	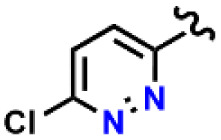	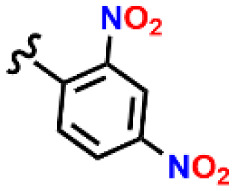	220, 228
Br	Me	Me	217c, 224c	F	n.a.	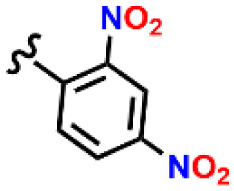	227a
Me	Me	Me	217d, 224d	Cl	n.a.	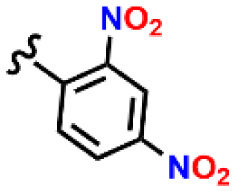	227b
F	Bn	Bn	218a, 225a	Br	n.a.	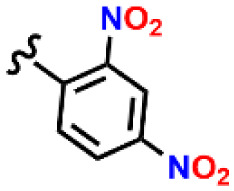	227c
Cl	Bn	Bn	218b, 225b	Me	n.a.	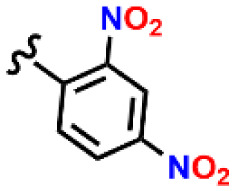	227d
Br	Bn	Bn	218c, 225c				

**Scheme 43:**
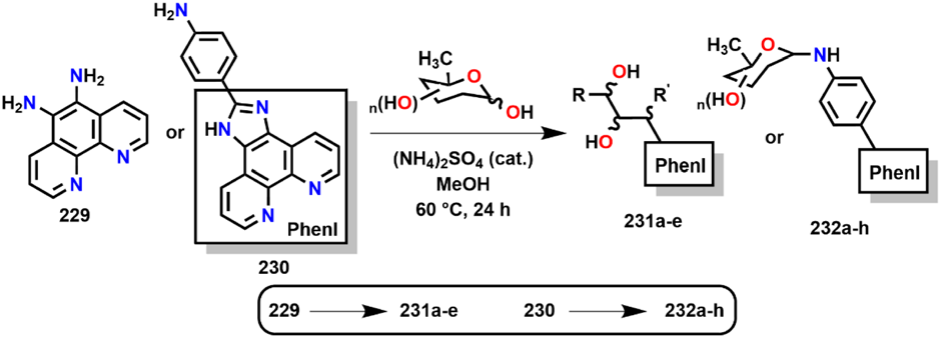
Synthesis of carbohydrate functionalized 1,10-phenanthrolines.

While the method utilizing ammonium acetate in acetic conditions was the most prominent to yield the imidazole-fused 1,10-phenanthroline, other possibilities were described as well. An interesting pathway was investigated by Gómez-Bra *et al.*, by using aldohexoses and -pentoses, the condensation reaction could be achieved with 5,6-diamino-1,10-phenanthroline (229) as the starting material (Scheme 43). A condensation reaction between the glycosidic hydroxyl function and one of the amine moieties of 1,10-phenanthroline is involved in the proposed mechanism, thus achieving an imine. The imidazole ring formation is catalyzed by the solvent methanol, promoting a favored cyclisation, achieving 231a–e.^104^ This mechanism was also utilized, albeit only partly, by Gratal *et al.* to synthesize glycoside derivatives of 1,10-phenanthroline (Scheme 43). However, the imidazole formation wasn't achieved during reactions as compound 230 was used as a starting material by Gratal *et al.*, which already featured said function. Without the presence of the second amine moiety, a different route was taken by the reaction, allowing the formation of the desired aminoglycoside derivatives (232a–e) (Table 12).^105^

**Table 12:** Definition of glycoside function described in Scheme 43

Glycoside	Compound	Glycoside	Compound
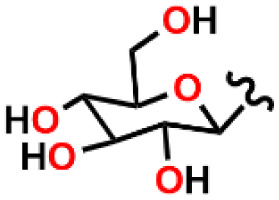	231a	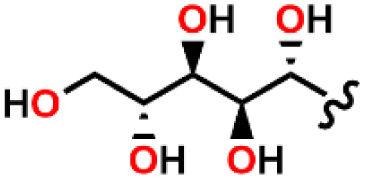	232c
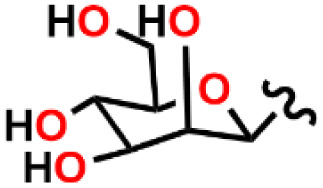	231b	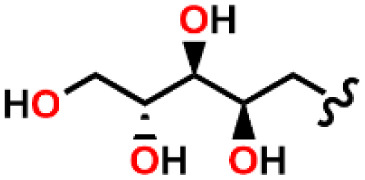	232d
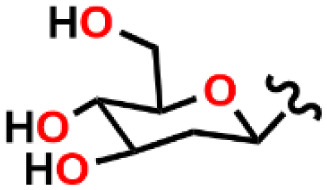	231c	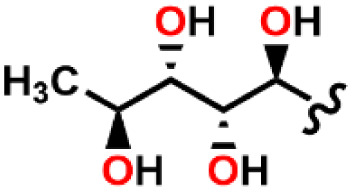	232e
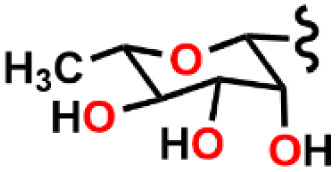	231d	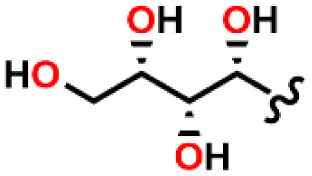	232f
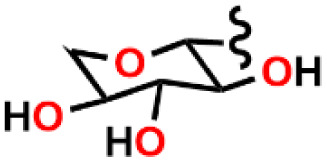	231e	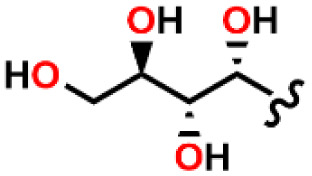	232g
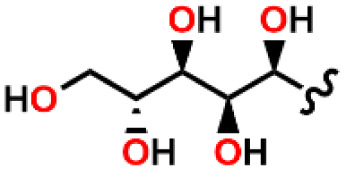	232a	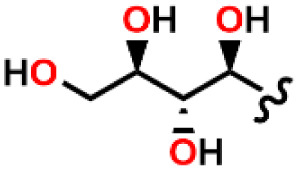	232h
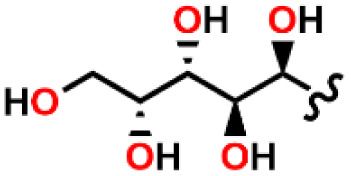	232b		

**Scheme 44:**
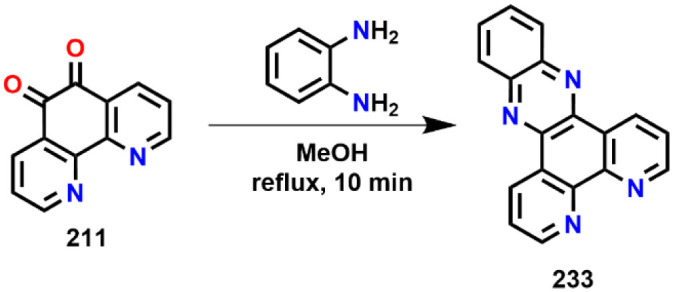
Synthesis of quinoxaline fused derivative.

**Scheme 45:**
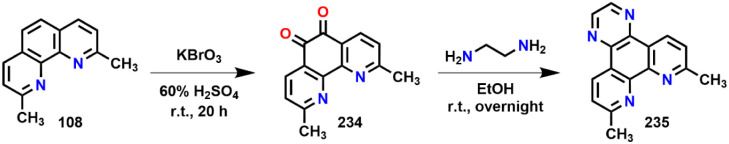
Synthesis of pyrazine fused derivative.

As the imidazole fused 1,10-phenanthroline derivative was becoming more prominent as having potent Gq affinity other ring systems were investigated as candidates to be condensed with 1,10-phenanthroline. One of the first to be synthesized is the quinoxaline fused derivative reported by Dupureur *et al.* and later used by Sun *et al.* as ligands for ruthenium complexation (Scheme 44). 1,10-Phenanthroline-5,6-dione is reacted with *o*-phenylene diamine in a simple condensation reaction to acquire 233 as the product with acceptable yields.^106,107^ This method was later utilized by Figueiredo *et al.* to achieve the synthesis of a pyrazine condensed analogue 235 from ethylene diamine (Scheme 45). Slight modifications were employed by Sun *et al.* as the solvent was changed from methanol to ethanol and the reaction was performed at r.t. instead of reflux temperatures.^108^ Due to these alterations, similar yields were achieved as described by Dupureur *et al.*

**Scheme 46:**
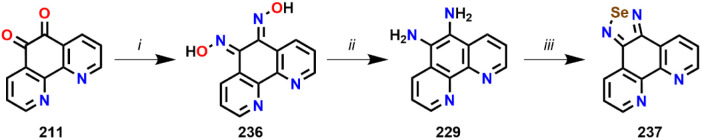
Synthesis of selenium fused derivative. (i) NH_2_OH·HCl, BaCO_3_, EtOH; (ii) Pd/C, EtOH; (iii) SeO_2_, H_2_O, r.t.

A unique approach was taken by Li *et al.* in the extension of Gq binding fused 1,10-phenanthroline molecular library. The first synthesis of a selenium heterocycle condensed 1,10-phenanthroline analogue was described by the research group (Scheme 46). Starting from 211 and through a two-step reaction involving the synthesis of bis-oxime derivative (236) and its subsequent reduction 1,10-phenanthroline-5,6-diamine (229) was achieved. The selenium heterocycle 237 was formed from 229 using selenium dioxide at r.t.^109^

**Scheme 47:**
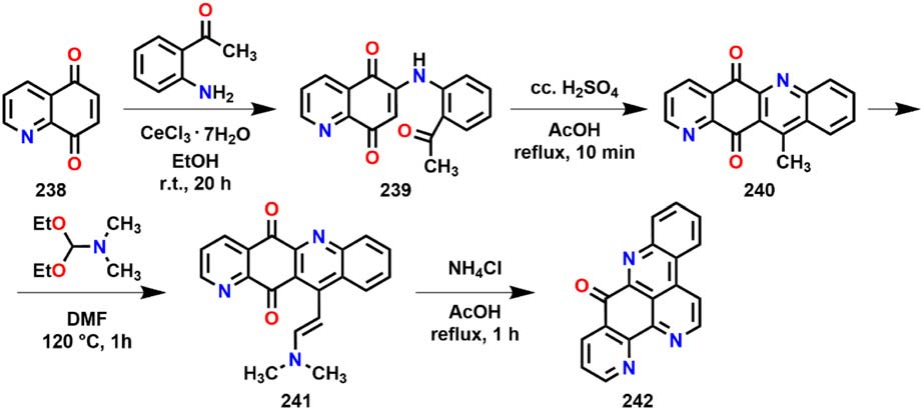
Total synthesis of ascididemin described by Bracher.

An interesting fused 1,10-phenanthroline derivative is the naturally occurring alkaloid ascididemin, containing a quinoline ring condensed to the anellation of the tricycle. It was first isolated from *Didemnum* species with rather low yields.^110^ This warranted a total synthetic method to produce the compound. Bracher achieved the synthesis in 1989 from quinoline-5,8-quinone in a 4-step reaction (Scheme 47). 238 was reacted with *o*-aminoacetophenone in the presence of cerium(iii)-chloride as a catalyst. The resulting secondary amine-linked molecule 239 was treated with concentrated sulfuric acid to achieve the ring closure and compound 240. Its reaction with DMF-diethyl acetal yielded enamine derivative 241, which after using ammonium chloride under acidic conditions, transformed into the desired ascididemin (242). The overall 40% yield was due to the last two steps, performing with much lower efficiency.^111^

**Scheme 48:**
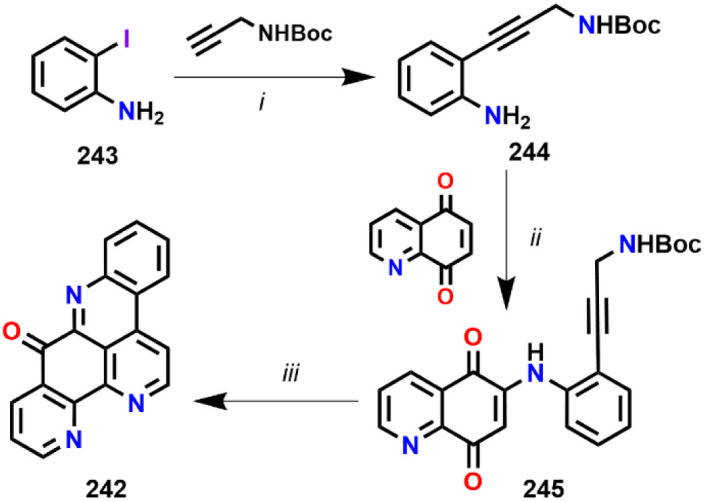
Total synthesis of ascididemin described by Yin *et al.* and Wumaier *et al.* (i) PdCl_2_(PPh_3_)_2_, CuI, TEA, r.t., 12 h; (ii) O_2_, CeCl_3_·7H_2_O, MeOH, r.t., 24 h; (iii) Fe_2_(SO_4_)_3_, H_2_SO_4_/AcOH, 100 °C, 2 h.

Later research revealed the potential of ascididemin to bind to Gq structures;^112^ thus a different approach was investigated by Yin *et al.* and Wumaier *et al.* to improve yields of the total synthetic method (Scheme 48). *o*-Iodoaniline (243) was utilized as a starting material, which was reacted with a protected propargyl amine to substitute the iodine, yielding 244. The following step is similar to the first step of the procedure described by Bracher (Scheme 47), the only difference being the presence of an oxygen atmosphere and a solvent exchange. To achieve 242, 245 was then reacted in the presence of iron(iii)-sulphate and sulfuric acid, finishing the formation of the 1,10-phenanthroline ring. The overall yield of ascididemin was improved by this method to around 50% at the cost of using a less readily available catalyst. Utilizing this procedure, other substituted ascididemin derivatives could also be achieved as well.^113,114^

## 4 Conclusions

With the discovery of their potential use as telomerase inhibitors acridine and 1,10-phenanthroline derivatives have undergone major structural changes to enhance their capability to intercalate into Gq complexes. Judging from the reviewed literature the most prominent of these modifications is the amide formation reactions that can be found in nearly all derivatives. Based on the established principles mentioned in the introduction, planar structures are proven to be beneficial at enhancing the Gq binding properties of molecules. When introducing such functions, an amide linker could be the best choice as it also has such a property. Also, based on the literature, extensive branching amide side-chains, further featuring aromatic ring systems, seem to be favoured by research groups in the development of novel Gq binders. Another principle that can enhance the biological activity of these molecules is the inclusion of cationic centers. Secondary, tertiary, and quaternary amine moieties are thus a prominent function when the modelling of novel acridine and 1,10-phenanthroline derivatives is considered. The formation of these functions was achieved using various methods, including nucleophilic substitutions and the reduction of the formed Schiff-bases. These reactions also opened the way to functionalize the acridine and 1,10-phenanthroline ring systems with further conjugated double-bonded systems, thus extending the π-π stacking interaction to the side-chains. A novel method also bearing the advantage of the aforementioned reactions, was also found. Utilizing the modified Mannich-reaction secondary and tertiary amine moieties can be formed using relatively readily available reagents in an effective way, while with carefully chosen reaction partners, other beneficial attributes *e.g.* π-π stacking interactions could be enhanced. This advantageous interaction can also be improved *via* condensation reactions, leading research groups to synthesize 1,10-phenanthroline analogues fused with heteroaromatic rings, including imidazole as the most prominent of these scaffolds.

While their potential as efficient Gq binders were highlighted, it should be noted that certain challenges can interfere with the development of novel acridine and 1,10-phenanthroline compounds. These ring systems due to the presence of heteroatoms are less electron dense, thus to achieve certain reactions the utilization of harsh reagents and conditions are a necessity. This can be a limiting factor in the synthesis of new compounds, while also posing safety and environmental risks as well. In future investigations regarding these compounds greener and safer alternatives should be explored. Another practical aspect that should be considered while working with these compounds is their potent complexing capabilities. Due to this attribute certain purification and practical methods involving the presence of metals are less effective in case of acridine and especially 1,10-phenanthroline. The toxicity of acridine and 1,10-phenanthroline compounds are also enhanced by their chelating capabilities. These scaffolds are regarded as highly carcinogenic due to their ability to intercalate into DNA, cleave nucleic acids and bind iron and copper ions in cells.^115,116^

The main guideline of acridine and 1,10-phenanthroline analogues was for a long time BRACO19 and PhenDC3, respectively leading to the investigation of symmetrical compounds by most research groups. This can hardly be called surprising as the nature of these molecules –being amides with extensive amide side-chains – can be attributed to their excellent biological activity, which is further enhanced by the symmetrical component. However, asymmetrical derivatives were also synthesized in later works and, while the literature of these compounds is still not as broad as the symmetrical analogues, they were found to be competent Gq binders. This finding also led the way to the synthesis of other angular condensed ring systems (*e.g.* benzo[*h*]quinoline), thus opening the way to the investigation of other 1,10-phenanthroline isostere compounds as potential Gq binders.

## Author contributions

Conceptualization, I. S., B. L.; investigation, S. J. R.; writing – original draft preparation, S. J. R.; writing – review and editing, I. S., B. L. All authors have read and agreed to the published version of the manuscript.

## Conflicts of interest

There are no conflicts to declare.

## Data Availability

This review compiles and analyses data from previously published studies on acridine- and 1,10-phenanthroline-based G-quadruplex binding compounds. No new experimental data were generated. All data are available within the article and its cited references.
